# The Impact of Individual Anthropogenic Emissions Sectors on the Global Burden of Human Mortality due to Ambient Air Pollution

**DOI:** 10.1289/EHP177

**Published:** 2016-05-13

**Authors:** Raquel A. Silva, Zachariah Adelman, Meridith M. Fry, J. Jason West

**Affiliations:** Department of Environmental Sciences and Engineering, University of North Carolina at Chapel Hill, Chapel Hill, North Carolina, USA

## Abstract

**Background:**

Exposure to ozone and fine particulate matter (PM2.5) can cause adverse health effects, including premature mortality due to cardiopulmonary diseases and lung cancer. Recent studies quantify global air pollution mortality but not the contribution of different emissions sectors, or they focus on a specific sector.

**Objectives:**

We estimated the global mortality burden of anthropogenic ozone and PM2.5, and the impact of five emissions sectors, using a global chemical transport model at a finer horizontal resolution (0.67° × 0.5°) than previous studies.

**Methods:**

We performed simulations for 2005 using the Model for Ozone and Related Chemical Tracers, version 4 (MOZART-4), zeroing out all anthropogenic emissions and emissions from specific sectors (All Transportation, Land Transportation, Energy, Industry, and Residential and Commercial). We estimated premature mortality using a log-linear concentration–response function for ozone and an integrated exposure–response model for PM2.5.

**Results:**

We estimated 2.23 (95% CI: 1.04, 3.33) million deaths/year related to anthropogenic PM2.5, with the highest mortality in East Asia (48%). The Residential and Commercial sector had the greatest impact globally—675 (95% CI: 428, 899) thousand deaths/year—and in most regions. Land Transportation dominated in North America (32% of total anthropogenic PM2.5 mortality), and it had nearly the same impact (24%) as Residential and Commercial (27%) in Europe. Anthropogenic ozone was associated with 493 (95% CI: 122, 989) thousand deaths/year, with the Land Transportation sector having the greatest impact globally (16%).

**Conclusions:**

The contributions of emissions sectors to ambient air pollution–related mortality differ among regions, suggesting region-specific air pollution control strategies. Global sector-specific actions targeting Land Transportation (ozone) and Residential and Commercial (PM2.5) sectors would particularly benefit human health.

**Citation:**

Silva RA, Adelman Z, Fry MM, West JJ. 2016. The impact of individual anthropogenic emissions sectors on the global burden of human mortality due to ambient air pollution. Environ Health Perspect 124:1776–1784; http://dx.doi.org/10.1289/EHP177

## Introduction

Rising anthropogenic emissions of air pollutants and their precursors have significantly increased ambient air pollution in many parts of the world (Cooper et al. 2014; Lamarque et al. 2010; Naik et al. 2013; Stevenson et al. 2013). Ozone and fine particulate matter (PM_2.5_) are particularly important for public health. Short-term exposure to ozone is associated with respiratory morbidity and mortality (Bell et al. 2014; Gryparis et al. 2004; Levy et al. 2005; Stieb et al. 2009), and long-term exposure has been linked to premature respiratory mortality in adults (Jerrett et al. 2009) and to increased risk of death in susceptible populations with chronic cardiopulmonary diseases and diabetes (Zanobetti and Schwartz 2011). Exposure to PM_2.5_ can have detrimental acute and chronic health effects, including premature mortality due to cardiopulmonary diseases and lung cancer (Brook et al. 2010; Burnett et al. 2014; Hamra et al. 2014; Krewski et al. 2009; Lepeule et al. 2012).

The global burden of disease (GBD) due to ambient air pollution was first estimated for urban PM_2.5_ based on surface measurements (Cohen et al. 2004). More recent studies have included urban and rural regions, using output from global atmospheric models (Anenberg et al. 2010; Fang et al. 2013; Lelieveld et al. 2013, 2015; Rao et al. 2012) or global modeling output combined with observations (Evans et al. 2013; Lim et al. 2012) to estimate exposure to PM_2.5_ and ozone. Our research group previously used output from an ensemble of global chemistry–climate models to estimate 2.1 million premature deaths/year associated with anthropogenic PM_2.5_ and 470,000 deaths/year associated with ozone (Silva et al. 2013).

Here, we have used a global chemical transport model at fine horizontal resolution to estimate the impact of removing anthropogenic emissions from each of five sectors (Energy, Residential and Commercial, Industry, Land Transportation, and Shipping and Aviation) on the global and regional mortality burden of anthropogenic ozone and PM_2.5_.

Understanding the impact of different sectors on the global burden and the relative importance of each sector among regions can help prioritize national and international air pollution control strategies. Although the impact of different sectors on health has been quantified in the United States (Caiazzo et al. 2013; Fann et al. 2013), Europe (Andersson et al. 2009; Brandt et al. 2013) and, very recently, globally (Lelieveld et al. 2015), other previous global studies have focused on one sector—Shipping (Corbett et al. 2007), Aviation (Barrett et al. 2010), or Land Transportation (Global Road Safety Facility, The World Bank; Institute for Health Metrics and Evaluation 2014; Chambliss et al. 2014). Using output from the same baseline and land transportation simulations as those used in the present study, Chambliss et al. (2014) calculated the fraction of total PM_2.5_ concentrations attributable to surface transportation emissions, applied that to the total PM_2.5_ concentrations determined by Brauer et al. (2012) to obtain country-level attributable fractions, and applied those fractions to the GBD 2010 national mortality estimates (Lim et al. 2012).

Estimates of health impacts using output from global models are limited by coarse model resolution that cannot resolve fine gradients in air pollutant concentrations. Coarse resolution estimates are expected to underestimate PM_2.5_-related mortality, mostly because of smoothing of high urban concentrations, with smaller bias for ozone-related mortality (Li et al. 2016; Punger and West 2013). We attempted to minimize these errors by performing simulations at a finer horizontal resolution (0.67° × 0.5°) than previous global modeling studies assessing health impacts (1° × 1° to 2.8° × 2.8°). In addition, we have quantified the bias in mortality estimates by comparing our results with those obtained using simulations at coarser resolution.

## Methods

### Modeled Ozone and PM_2.5_ Concentrations

We simulated ozone and PM_2.5_ concentrations for 2005 using the Model for Ozone and Related Chemical Tracers, version 4 (MOZART-4). MOZART-4 includes a chemical mechanism with detailed hydrocarbon chemistry and bulk aerosols, as well as online representations of several processes such as dry deposition, biogenic emissions of isoprene and monoterpenes, and photolysis frequencies (Emmons et al. 2010). Anthropogenic and biomass burning emissions are from the Representative Concentration Pathway 8.5 global emissions inventory for 2005 (Riahi et al. 2011) (see Supplemental Material, “Input emissions” and Tables S1, S2). Biogenic emissions of isoprene and monoterpenes were calculated online in MOZART-4 using the Model of Emissions of Gases and Aerosols from Nature (MEGAN) (Guenther et al. 2006). All other natural emissions were taken from Emmons et al. (2010). The model was run at a 0.67° longitude by 0.5° latitude horizontal resolution with 72 vertical hybrid (sigma and pressure) levels driven by GEOS-5 meteorological fields. Each simulation was run for 18 months, including 6 months spin-up. Surface concentrations were from the lowest vertical level (992.5 mb at the layer midpoint).

Simulated 2005 surface concentrations show similar agreement with observations to that of other global models (see Supplemental Material, “MOZART-4 performance evaluation” and Figures S1–S6), and to that of previous MOZART-4 simulations at a coarser resolution using the same meteorology and emissions inputs (Fry et al. 2013). Additionally, we ran a simulation with no anthropogenic emissions to estimate the total mortality burden of present-day anthropogenic ozone and PM_2.5_ (see Supplemental Material, “Simulation with zeroed-out anthropogenic emissions”). Both simulations were also run at a coarser resolution (2.5° × 1.9°) to estimate the bias relative to the fine resolution.

The impact of removing emissions from each source sector was quantified using a brute-force sensitivity analysis, in which five emissions sectors were zeroed out individually: All Transportation, Land Transportation, Energy, Industry, and Residential and Commercial. Land Transportation is a subset of All Transportation; we estimated the impact of Shipping and Aviation as the difference. This zero-out method has been used in previous studies to evaluate the contribution of different regions and/or sectors to ambient air pollutant concentrations (e.g., Andersson et al. 2009; Caiazzo et al. 2013; Corbett et al. 2007; Koch et al. 2007; Li et al. 2016). Because of nonlinearity in the model’s response to changes in emissions (e.g., emission reductions may change the ozone chemical regime), estimates of the impacts of a sector using the zero-out method may differ from those obtained by other methods (e.g., source tracking), and the sum of source sector impacts may differ from the total in the baseline simulation (Cohan et al. 2005; Koo et al. 2009; Kwok et al. 2015).

Modeled concentrations in each grid cell were processed to obtain the metrics used in the health impact assessment, consistent with the underlying epidemiological studies (Burnett et al. 2014; Jerrett et al. 2009; Krewski et al. 2009): annual average PM_2.5_ and average 1-hr daily maximum ozone for the consecutive 6-month period with the highest average. PM_2.5_ concentrations were estimated as a sum of modeled species (see Supplemental Material, “Ozone and PM_2.5_ surface concentrations” and Tables S3–S5, Figures S7–S12).

### Health Impact Assessment

We estimated cause-specific excess mortality due to exposure to ambient air pollution (*ΔMort*) in each MOZART-4 grid cell as *ΔMort = y_0_* × *AF* × *Pop*, where *y_0_* is the baseline mortality rate (for the exposed population), *AF = 1–1/RR* is the attributable fraction (*RR* = relative risk of death attributable to a change in pollutant concentration), and *Pop* is the exposed population (adults ≥ 25 years old).

For ozone, *AF = 1 – exp^–^* ^β^ *^ΔX^*, where *RR = exp* ^β^ *^ΔX^*, β is the concentration–response factor, *ΔX* corresponds to the change in pollutant concentrations, and *RR* = 1.040 [95% confidence interval (CI): 1.013, 1.067] for a 10 ppb increase in ozone concentrations according to Jerrett et al. (2009), who performed the largest study to date to estimate *RR* for long-term exposure to ozone. Although Jerrett et al. (2009) estimated *RR* for adults ≥ 30 years old, we considered adults ≥ 25 years old, assuming identical *RR*, to align exposed populations for ozone and PM_2.5_, following the method used by Lim et al. (2012). However, we evaluated ozone mortality due to all chronic respiratory diseases (World Health Organization, *International Classification of Diseases*, *9th revision;* ICD-9 BTL: B347) based on Jerrett et al. (2009), as other global studies have done (Anenberg et al. 2010; Fang et al. 2013; Lelieveld et al. 2013; Silva et al. 2013), whereas Lim et al. (2012) considered only chronic obstructive pulmonary disease (COPD) mortality (78% of global chronic respiratory disease mortality, ranging from 27% to 93% nationally).

For PM_2.5_, we used the integrated exposure–response (IER) model developed for GBD 2010 (Burnett et al. 2014), which is intended to provide better estimates of mortality than other models at high PM_2.5_ concentrations:


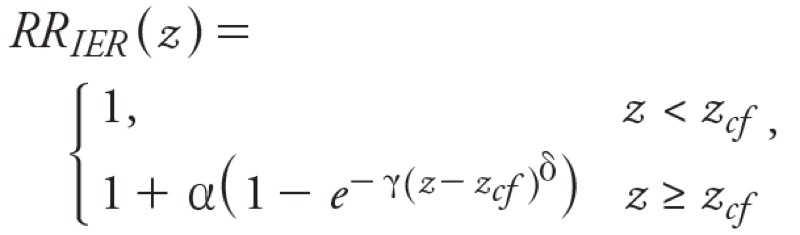
[1]

where *z* is PM_2.5_ concentration and *z_cf_* is the counterfactual concentration (theoretical minimum-risk exposure, assumed by Burnett et al. (2014) to have a uniform distribution: *z_cf_ ~ U*[5.8,8.8]).

We used the RRs given by IER for mortality due to ischemic heart disease (IHD; ICD-9: 410–414), cerebrovascular disease (Stroke; ICD-9: 430–435, 437.0–437.2, 437.5–437.8), COPD (ICD-9: 490–492.8, 494, 496), and lung cancer (LC; ICD-9 BTL: B101). We used the values for parameters α, γ, and δ reported by Burnett et al. (2014) for 1,000 simulations [Global Health Data Exchange (GHDx) 2013]. We calculated *AF = AF_1_ – AF_2_*, where *AF_1_ = 1 – 1/RR_IER(z1)_* and *AF_2_ = 1 – 1/RR_IER(z2)_*, *z_1_* = baseline concentration (simulation with all anthropogenic emissions), *z_2_* = concentration in control simulation (with zeroed-out emissions).

We defined the mortality burden of anthropogenic air pollution as that which is controllable, using the simulation with no anthropogenic emissions to estimate *ΔX* for ozone and *z_2_* for PM_2.5_, following the approach used by Anenberg et al. (2010), Fang et al. (2013), Lelieveld et al. (2013), and Silva et al. (2013). This approach differs from that of GBD 2010, which considered total PM_2.5_ relative to *z_cf_* (*AF = AF_1_*). Where natural PM_2.5_ ≤ *z_cf_* (*AF_2_* = 0), our estimate of excess mortality is identical to the total PM_2.5_ mortality burden. If natural PM_2.5_ concentrations > *z_cf_* (e.g. dusty regions), we estimate mortality due to anthropogenic air pollution only. Whereas Giannadaki et al. (2014) quantified the contribution of desert dust to global mortality, it is considered natural PM_2.5_ under our definition. In addition, given the nonlinearity of the IER model, we assumed that the impact of removing each sector corresponded to the difference in mortality estimates for PM_2.5_ concentrations in each zeroed-out simulation relative to the total PM_2.5_. As a sensitivity analysis, we also used the log-linear function with RR for CPD and LC from the report of Krewski et al. (2009), following other global health assessments (Anenberg et al. 2010; Evans et al. 2013; Fang et al. 2013; Lelieveld et al. 2013; Silva et al. 2013).

Exposed population was obtained from the Oak Ridge National Laboratory’s LandScan 2011 Global Population data set at approximately 1 km resolution (30˝ × 30˝) (Bright et al. 2012). For adults ≥ 25 years old, we estimated the population per 5-year age group in each cell by multiplying the country-level percentage in each age group (from LandScan) by the total cell population using ArcGIS 10.2. Cause-specific baseline mortality rates for 187 countries were obtained from the GBD 2010 mortality data set [Institute for Health Metrics and Evaluation (IHME) 2013]. We estimated the number of deaths per 5-year age group per country using the national population from LandScan and gridded these values using ArcGIS 10.2. The resulting population and baseline mortality per age group at 30˝ × 30˝ were regridded to the resolutions of the atmospheric model (0.67° × 0.5° and 2.5° × 1.9°).

We conducted 1,000 Monte Carlo (MC) simulations to propagate uncertainty from the RRs, baseline mortality rates, and modeled air pollutant concentrations using random sampling of the three variables simultaneously. For ozone RRs, we used the reported 95% CIs and assumed a normal distribution. For PM_2.5_ RRs, we used the parameter values of Burnett et al. (2014) for 1,000 simulations (GHDx 2013). In addition, we considered the reported 95% CIs for baseline mortality rates, assuming lognormal distributions. Finally, for modeled ozone and PM_2.5_ concentrations, we used the absolute value of the coefficient of variation (= standard deviation/mean) at each grid cell for the year 2000 minus year 1850 simulations from the Atmospheric Chemistry and Climate Model Intercomparison Project (ACCMIP) ensemble (Lamarque et al. 2013; Silva et al. 2013), regridded to 0.67° × 0.5° and following a normal distribution. Uncertainty associated with the population was assumed to be negligible. For each MC simulation, we obtained the regional and global totals, which we then used to estimate the empirical mean and 95% CI of the regional and global mortality results. We estimated the contribution of uncertainty in each variable to overall uncertainty in mortality estimates using a tornado analysis.

## Results

Global ozone and PM_2.5_ surface concentrations and population-weighted averages for 10 world regions, exposed population, and baseline mortality rates are shown in the Supplemental Material, “Ozone and PM_2.5_ surface concentrations” (see also Figures S7–S12) and “Population and Baseline Mortality Rates” (see also Table S6).

**Figure 1:**
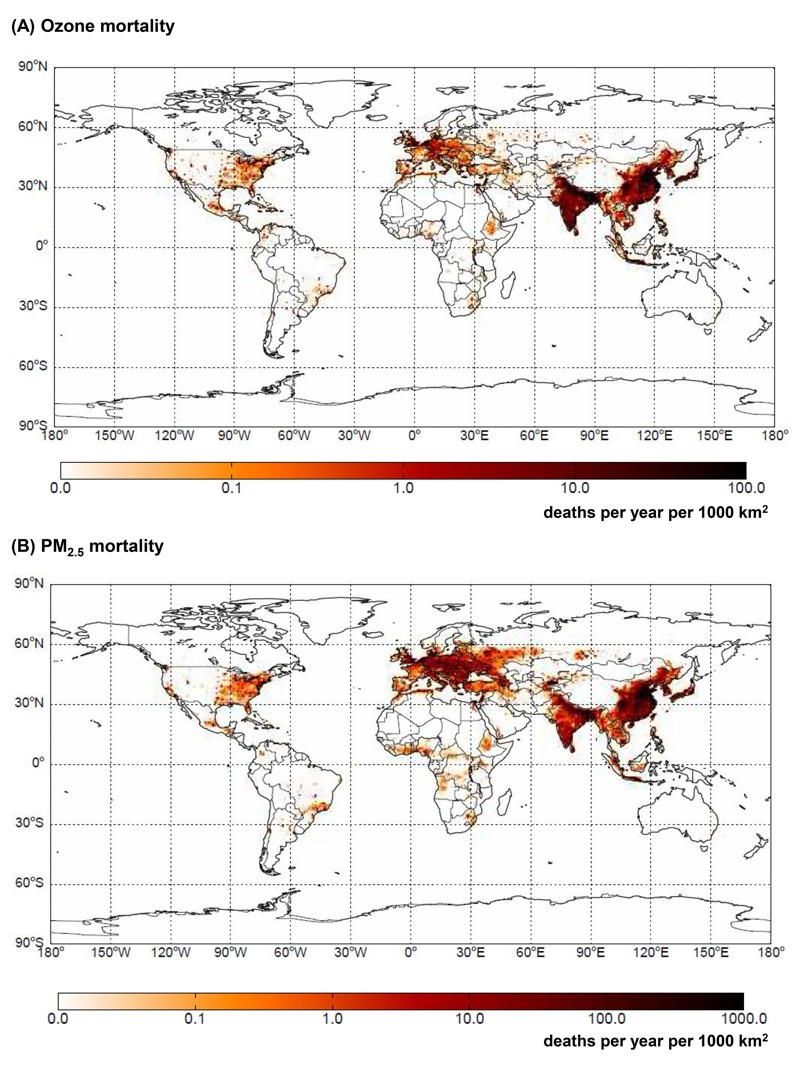
Premature ozone-related respiratory mortality (*A*) and PM_2.5_-related mortality [ischemic heart disease (IHD) + stroke + chronic obstructive pulmonary disease (COPD) + lung cancer (LC)] (*B*) in 2005 (deaths per year per 1,000 km^2^), shown as the mean of 1,000 Monte Carlo simulations.

We estimated the present-day global burden of anthropogenic ozone–related respiratory mortality to be 493 (95% CI: 122, 989) thousand deaths/year (Table 1). Most mortality occurred in East Asia (35%) and India (33%) (Figure 1; see also Tables S7, S8). These regions are highly populated and, together with North America, have the highest population-weighted average anthropogenic ozone concentrations. East Asia and India had 113 deaths/year per million people because of ozone, whereas the lowest premature mortality rate occurred in Africa (11 deaths/year per million people) (see Table S9). For global ozone mortality, the coefficient of variation (CV; standard deviation/mean) is 46%, and uncertainty in β and in *ΔX* have similar contributions to overall uncertainty (45% each), whereas uncertainty in *y_0_* contributes 10%.

**Table 1:** Global premature ozone and PM_2.5_-related mortality, and impact of removing emissions from individual sectors (thousand deaths in 2005), showing the mean and 95% confidence interval.

	All anthropogenic	All transportation	Land transportation	Energy	Industry	Residential and commercial
Ozone mortality	493 (122, 989)	115 (27.8, 244)	80.9 (17.4, 180)	65.2 (14.5, 143)	45.6 (8.7, 96.8)	53.7 (12.3, 116)
PM_2.5_ mortality	2,230 (1,040, 3,330)	261 (136, 364)	212 (114, 292)	290 (192, 386)	323 (230, 430)	675 (428, 899)

For anthropogenic PM_2.5_, we estimated a global mortality burden of 2.2 (95% CI: 1.0, 3.3) million deaths/year (Table 1), with contributions from IHD [926 (95% CI: 436, 1,300) thousand], stroke [887 (95% CI: 439, 1,300) thousand], COPD [260 (95% CI: 79.2, 477) thousand] and LC [157 (95% CI: 29.8, 316) thousand]. The greatest mortality occurred in East Asia (48%), followed by India (18%) and Europe (11%) (Figure 1; see also Tables S10, S11), regions with the highest population-weighted average anthropogenic PM_2.5_. The number of deaths in Australia and South America was very low owing to large areas with low population density; in addition, these regions had the lowest average PM_2.5_ concentrations (see Table S4), which were below the threshold of the IER function in many grid cells. East Asia has 683 deaths/year per million people due to anthropogenic PM_2.5_, and the lowest mortality rate occurs in Africa (32 deaths/year per million people) (see Table S12). The global CV for PM_2.5_ mortality was 25%, but global CVs were greater for COPD (40%) and LC (46%) than for IHD (25%) and stroke (26%). Uncertainties in the RR model parameters α, γ and δ together had the greatest contribution to overall uncertainty (71.7%), followed by *z_1_* (23.3%), but *z_2_* (2.3%), *y_0_* (2.4%), and *z_cf_* (0.2%) contributed little to overall uncertainty. When each disease was considered individually, the contributions of different variables varied from those mentioned above, particularly the contributions of *z_1_* to IHD (33.2%), COPD (14.1%), and LC (13.0%) mortality uncertainties.

**Figure 2:**
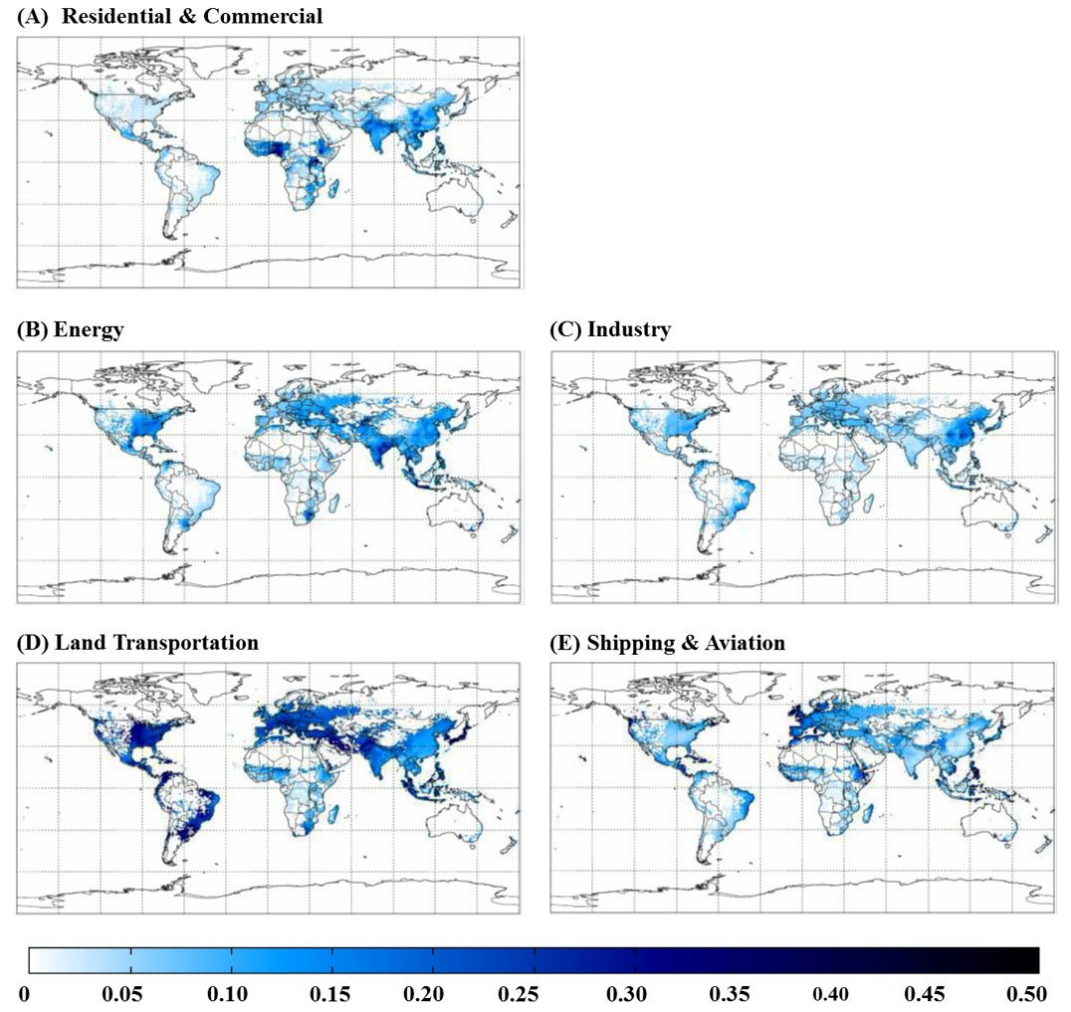
Impact of removing emissions from each sector (*A*–*E*) on total ozone-related respiratory mortality in 2005, shown as a ratio to the total burden in each cell. Areas shown as white have < 1 ozone-related death per grid cell.

**Figure 3:**
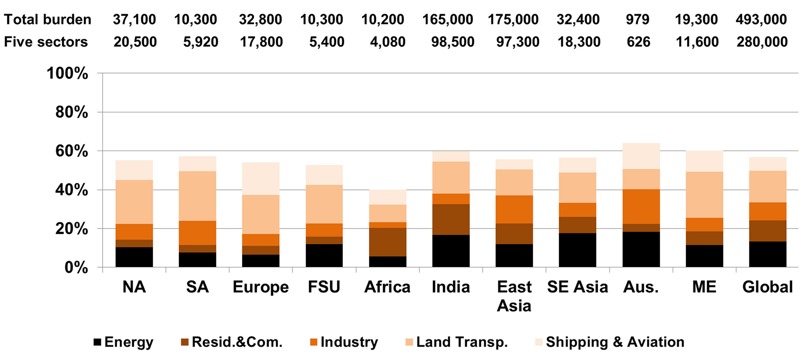
Impact of removing emissions from each sector on premature ozone-related respiratory mortality in each region and globally, relative to the total burden (deaths in 2005). Numbers above each column correspond to the total burden (all anthropogenic emissions zeroed out) and to the sum of the five sectors. Land Transp., Land Transportation; Resid. & Com., Residential and Commercial. The 10 world regions are defined in Figure S7: NA–North America, SA–South America, Europe, FSU–former Soviet Union, (Sub-Saharan) Africa, India, East Asia, SE Asia–Southeast Asia, Aus.–Oceania, ME–Middle East (and North Africa).

Globally, the zeroed-out sectors contributed ~57% of total anthropogenic ozone mortality (Table 1). Land Transportation had the greatest global impact (16%) and the greatest regional impact (20–26%) in North America, South America, Europe, FSU and the Middle East (Figures 2 and 3) because it strongly influences ozone concentrations. The Energy and Residential and Commercial sectors also had strong impacts in India, and all sectors had important impacts in East Asia. Among the deaths caused by each sector worldwide, the greatest impacts occurred in India and East Asia, particularly for Residential and Commercial (83%), Industry (75%), and Energy (74%), reflecting the large exposed populations in these regions. Within each region, there was variability in the impact of different sectors, with a few hotspots for certain sectors (e.g., central Africa for Residential and Commercial, eastern North America and India for Energy, and eastern East Asia for Industry). The 43% of the total burden not accounted for by the five modeled sectors likely reflects sectors that were not zeroed out, mainly Biomass Burning emissions, increases in methane from preindustrial times until the present day, and nonlinear model responses.

**Figure 4:**
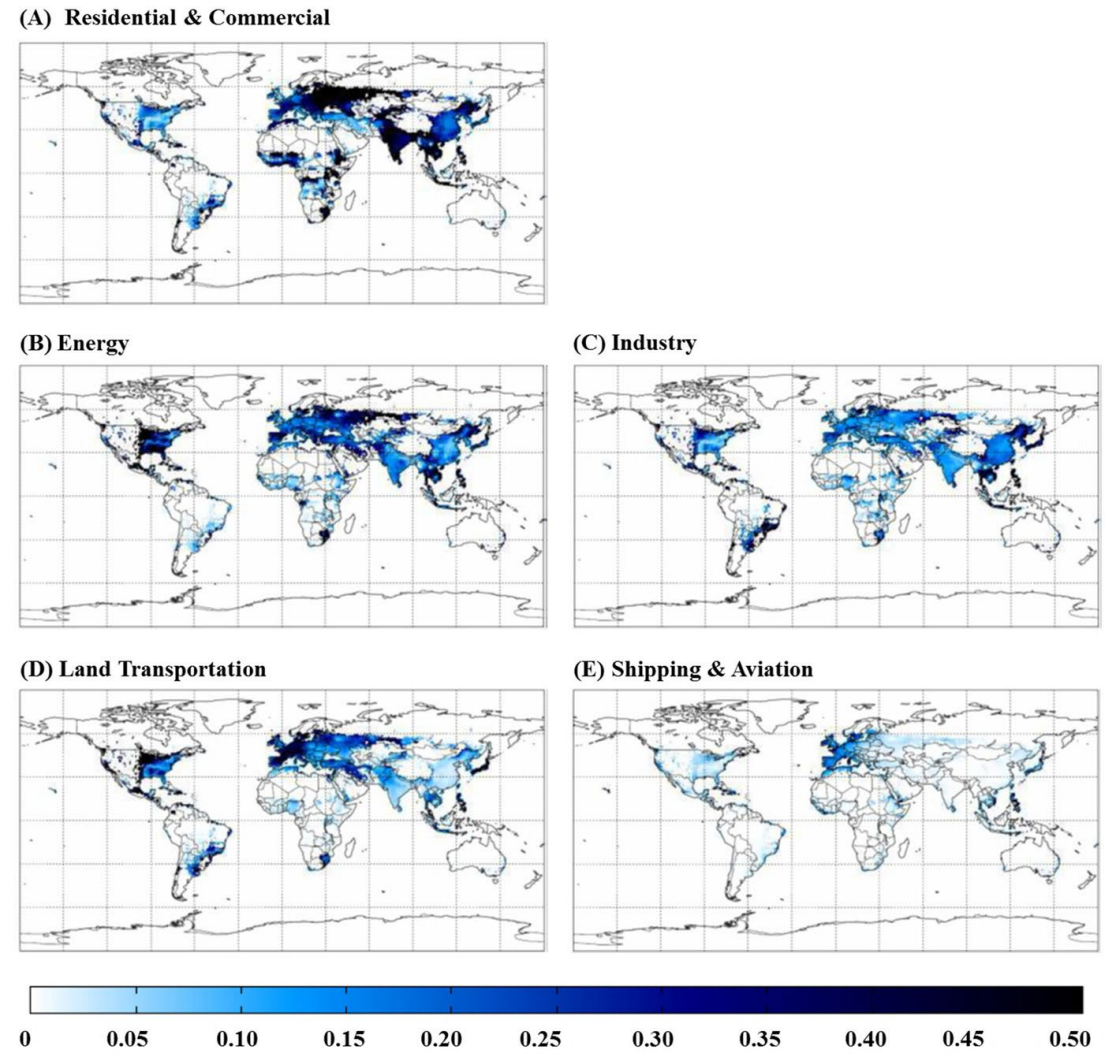
Impact of removing emissions from each sector (*A*–*E*) on total premature PM_2.5_-related mortality [ischemic heart disease (IHD) + stroke + chronic obstructive pulmonary disease (COPD) + lung cancer (LC)] in 2005, shown as the ratio of total burden in each cell. Areas shown as white have < 1 PM_2.5_-related death per grid cell.

**Figure 5:**
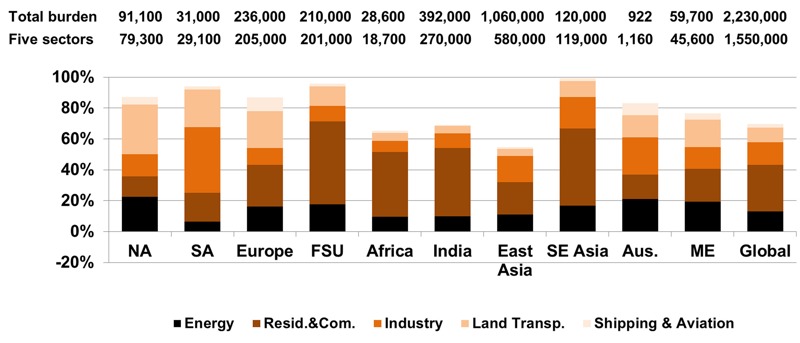
Impact of removing emissions from each sector on premature PM_2.5_-related mortality [ischemic heart disease (IHD) + stroke + chronic obstructive pulmonary disease (COPD) + lung cancer (LC)] in each region and globally, relative to the total burden (deaths in 2005). Numbers above each column correspond to the total burden (all anthropogenic emissions zeroed-out), and to the sum of the five sectors. Land Transp., Land Transportation; Resid. & Com., Residential and Commercial. The 10 world regions are defined in Figure S7: NA–North America, SA–South America, Europe, FSU–former Soviet Union, (Sub-Saharan) Africa, India, East Asia, SE Asia–Southeast Asia, Aus.–Oceania, ME–Middle East (and North Africa).

For anthropogenic PM_2.5_, the modeled sectors contributed 70% of total global mortality (Table 1). The Residential and Commercial sector contributed 675 (95% CI: 428, 899) thousand deaths/year, having the greatest impact globally (30%) and in most regions except North America, South America and Australia (Figures 4 and 5). Land Transportation dominated in North America (32% of total anthropogenic PM_2.5_ mortality in this region), and in Europe it had nearly the same burden (24%) as Residential and Commercial (27%). In East Asia, Residential and Commercial contributed 21% of total mortality, followed by Industry (17%) and Energy (11%). Residential and Commercial has the greatest impact in East Asia (33%), followed by India (26%). Industry and Energy also affected East Asia the most (55% and 41%, respectively). Land Transportation had the strongest impact in Europe (27%) and in East Asia (23%). The different regional impacts are associated with the effect of removing emissions from each sector on total anthropogenic PM_2.5_ concentrations and with the exposed population and baseline mortality rates in each region (e.g., cardiovascular diseases in FSU). The impact of each sector varied within each region, reflecting the location of emission sources (e.g., eastern North America for Energy; small areas in Europe, FSU, southern Africa, eastern South America, Middle East and East Asia for Energy and Industry). The 30% of the total burden not accounted for by the five modeled sectors is likely associated mainly with Biomass Burning emissions.

### Sensitivity Analyses

***Fine versus coarse resolution.*** Using output from simulations at fine and coarse grid resolutions to directly estimate mortality, we quantified a slight negative bias of 2% for global ozone mortality and a positive bias of 16% for global PM_2.5_ mortality at coarse resolution relative to fine resolution (see Supplemental Material, “Fine vs. coarse resolution,” and Table S13). When we regridded fine resolution–modeled concentrations to the coarse resolution, following the method reported by Punger and West (2013), the negative bias of the global mortality estimates for regridded ozone concentrations slightly increased to 3% (relative to the original fine resolution), but the bias for PM_2.5_ changed sign to a negative bias of 8% (see Supplemental Material, “Fine vs. coarse resolution,” and Table S14). The biases for mortality estimates obtained at the original coarse resolution reflected the total effect of grid resolution on both modeled “chemistry” (e.g., Wild and Prather 2006) and “exposure” (the spatial alignment of population and concentration), whereas the biases estimated using concentrations regridded to coarse resolution only captured the effect of resolution on exposure. For ozone, our total bias is very close to the “exposure” bias, suggesting a minor effect of resolution on modeled chemistry. For PM_2.5_, our positive total bias at coarse resolution likely reflects a local effect of grid resolution on PM_2.5_ chemistry. Our “exposure” negative bias of 8% for PM_2.5_ is comparable to those estimated by Punger and West (2013) and by Li et al. (2016), showing the effect on mortality estimates of the spatial degradation of urban PM_2.5_ concentrations.

***Log-linear exposure–response function for PM_2.5._*** Using the log-linear model and RRs of Krewski et al. (2009), we obtained 74% of the global burden of anthropogenic PM_2.5_ mortality estimated with the IER function, with marked regional differences (e.g., for North America, the log-linear estimate was 16% higher than the IER estimate). We used the RR reported for CPD for IHD, stroke and COPD and the RR reported for LC to allow a straightforward comparison with the IER estimate. IHD and stroke mortality decreased by 60% and 57%, respectively, whereas COPD and LC mortality increased by 131% and 107%, respectively.

These differences can be explained by the nonlinear shape of the IER function (Burnett et al. 2014), which gives considerably different estimates of AF for identical changes in PM_2.5_ concentrations in areas with low versus high total PM_2.5_ concentrations, such as North America (8.5 μg/m^3^) and Middle East (27.8 μg/m^3^), with the latter being on the flatter part of the IER curves. Population-weighted average anthropogenic PM_2.5_ concentrations (2005 minus natural) for North America and Middle East were very close (7.1 and 7.2 μg/m^3^, respectively), as were the attributable fractions for CPD (8.2% and 8.3%, respectively) and LC (9.0% and 9.1%, respectively) when using the log-linear model. However, using the RRs from the IER model, AFs for IHD for North America were between 21% and 6% for all age groups, whereas for Middle East, they were between 5% and 3%; for LC they were 2.0% (North America) and 3.9% (Middle East) for adults ≥ 25 years old.

## Discussion

Our global burden estimates are comparable to those of Silva et al. (2013), who used an ensemble of global models, being 5% greater for ozone mortality and 6% greater for PM_2.5_ mortality, although here we used the IER model to estimate PM_2.5_ mortality. For ozone mortality, our results differ from those of Anenberg et al. (2010) (–30%), Lim et al. (2012) (+228%), Fang et al. (2013) (+31%), Lelieveld et al. (2013) (–36%), and Lelieveld et al. (2015) (+246%). For PM_2.5_, our estimates are lower than those of Anenberg et al. (2010) (–40%), Lim et al. (2012) (–30%), Evans et al. (2013) (–18%), and Lelieveld et al. (2015) (–19%), but higher than those of Lelieveld et al. (2013) (+2%) and Fang et al. (2013) (+40%). We do not suggest that our estimates are better than those from these studies, but we highlight differences between approaches, particularly our use of a fine-resolution model and our evaluation of anthropogenic air pollution through comparison with a simulation with no anthropogenic emissions.

Our lower estimates than those reported by Anenberg et al. (2010) may be related to the finer resolution (vs. 2.8° × 2.8°) and updates in MOZART-4 (vs. MOZART-2)but are likely a result of the use of different emissions data sets, different exposure–response functions for PM_2.5_, and updated population and baseline mortality rates. We used the same exposure–response functions for PM_2.5_ as Lim et al. (2012) and Lelieveld et al. (2015), but we estimated anthropogenic PM_2.5_ mortality, whereas those authors estimated total PM_2.5_ mortality; furthermore, Lelieveld et al. (2015) used a different exposure–response function for ozone, and both Lim et al. (2012) and Lelieveld et al. (2015) considered a low-concentration threshold for ozone mortality and baseline mortality rates for COPD only (whereas we considered all chronic respiratory diseases). Differences in the spatial distributions of pollutant concentrations and exposed population may also be important. The other studies were based on model output from different global models using different inputs and definitions of anthropogenic air pollution (Fang et al. 2013; Lelieveld et al. 2013) or were based on observations and model output of total pollutant concentrations (Evans et al. 2013); their health impact assessments used the log-linear exposure–response function for PM_2.5_ as well as different population and baseline mortality rates.

A major contribution from this study is estimating sectoral contributions to the total burden of anthropogenic air pollution on mortality globally and regionally. Our estimates of nearly 50,000 PM_2.5_-related deaths/year attributable to Shipping and Aviation are ~30% lower than the combined estimates of Corbett et al. (2007) for Shipping and Barrett et al. (2010) for Aviation but are within their confidence intervals. For Land Transportation, our estimate is 12% lower than that of Chambliss et al. (2014), reflecting the difference in methodologies despite the use of identical modeled PM_2.5_ concentrations. For sectors also evaluated by Lelieveld et al. (2015), our results for the sum of ozone and PM_2.5_-related mortality are lower for Residential and Commercial (–27%) and Energy (–24%) and higher for Land Transportation (+79%) and Industry (+63%); these differences should be attributed to the methodological differences mentioned above as well as to the underlying emission inventories.

We chose not to add ozone and PM_2.5_ mortality to avoid possibly double-counting respiratory mortality because we included PM_2.5_ mortality associated with COPD. However, we calculated ozone respiratory mortality using RRs from Jerrett et al. (2009), who controlled for PM_2.5_; therefore, double-counting should be negligible owing to different biological mechanisms associated with exposure to each pollutant (Anenberg et al. 2010). Our results assume that the same RRs apply worldwide, even though underlying health conditions and PM_2.5_ composition vary. The RR for ozone is based on results from a U.S. cohort (Jerrett et al. 2009), and the IER function for PM_2.5_ is based on studies in North America, Western Europe, and China (Burnett et al. 2014). In addition, we limited our study to adults ≥ 25 years old, which may have underestimated total and sectoral burdens. We reduced the potential for coarse resolution bias by conducting simulations at a fine horizontal resolution for a global chemical transport model; however, our results are still limited by resolution and cannot fully resolve fine concentration gradients, particularly near urban areas. For example, emissions from the Residential and Commercial sector occur where people live, and more detailed spatial analyses may suggest a greater relative impact for this sector. Our uncertainty estimates are wider than those of other studies, reflecting our use of the spread of modeled concentrations from the ACCMIP multimodel ensemble. These estimates of uncertainty do not account for uncertainty in emissions inventories (because the ensemble used identical emissions), nor for uncertainty in exposed population, which is likely small.

## Conclusions

We found regional differences in the relative importance of emissions sectors to ambient air pollution–related mortality. Globally, we estimated 493,000 deaths/year due to anthropogenic ozone and 2.2 million deaths/year due to anthropogenic PM_2.5_. Land Transportation had the greatest impact on ozone respiratory mortality (80,900 deaths/year, 16% of the global burden), whereas the Residential and Commercial sector contributed the most to PM_2.5_-related premature mortality (IHD + stroke + COPD + LC) (675,000 deaths/year, 30%).

In East Asia, Industry had the greatest impact on ozone mortality (14%) and also had a great impact on PM_2.5_ mortality (17%), following Residential and Commercial (21%). In India, Energy had the greatest impact on ozone mortality (17%), but the Residential and Commercial sector clearly dominated PM_2.5_ mortality (43%). In North America, Land Transportation had the greatest impact on both ozone (23%) and PM_2.5_ (55%) mortality.

Uncertainty in RR and in modeled ozone concentrations had similar contributions to overall uncertainty in ozone mortality, whereas uncertainty in RR had the greatest impact on total PM_2.5_ mortality and, in particular, on COPD and LC mortality. Future epidemiological research on the long-term effects of air pollution should aim to narrow the uncertainty in RR, particularly in developing nations worldwide. Future research should also focus on improving emissions inventories for air quality modeling and on reducing the bias in modeled air pollutant concentrations.

The relative impact of removing emissions from different sectors on anthropogenic ozone- and PM_2.5_-related mortality in different regions suggests that location-specific air pollution control policies are appropriate. However, the development of improved emission control technologies may be pursued globally. Global actions to reduce emissions of ozone precursors from Land Transportation would be particularly beneficial for public health, as would reducing PM_2.5_ emissions from the Residential and Commercial sector. In East Asia, additional air pollution control strategies addressing all sectors would considerably lessen global mortality. Focusing on the Energy sector and on PM_2.5_ emissions from Industry in India, and on PM_2.5_ emissions from Land Transportation in North America and Europe would yield the greatest benefits for health.

## Supplemental Material

(6.2 MB) PDF
