# Supplementary material for: The Impact of Individual Anthropogenic Emissions Sectors on the Global Burden of Human Mortality due to Ambient Air Pollution
Source: Environ Health Perspect. 2016 May 13;124(11):1776–84. doi: 10.1289/EHP177 (PMC5089880; doi:10.1289/EHP177)
Supplement: (6.2 MB) PDF [file EHP177.s001.acco.pdf]

Environ Health Perspect
DOI: 10.1289/EHP177

Editor's Note: *The tables have been reordered since Supplemental Material was originally published. The current order reflects the citations in the final published article.*

Note to readers with disabilities: *EHP* strives to ensure that all journal content is accessible to all readers. However, some figures and Supplemental Material published in *EHP* articles may not conform to 508 standards due to the complexity of the information being presented. If you need assistance accessing journal content, please contact ehp508@niehs.nih.gov. Our staff will work with you to assess and meet your accessibility needs within 3 working days.

Supplemental Material

The Impact of Individual Anthropogenic Emissions Sectors on the Global Burden of Human Mortality due to Ambient Air Pollution

Raquel A. Silva, Zachariah Adelman, Meridith M. Fry, and J. Jason West

Table of Contents

1. Input emissions
2. MOZART-4 performance evaluation
3. Simulation with zeroed-out anthropogenic emissions
4. Ozone and $PM_{2.5}$ surface concentrations
5. Population and Baseline Mortality Rates
6. Additional mortality results
7. Sensitivity Analysis (additional information)
References

Table S1 – Emissions source sectors included in the RCP8.5 global emissions inventory.

Table S2 – Global emissions per sector for six pollutants (Tg/yr) in the RCP 8.5 Global Emissions Inventory, 2005, and the total emissions for all 12 sectors and for the sectors modeled in zero-out simulations (and percentage of the modeled sectors in total emissions in parenthesis).
Table S3 – Ozone concentrations for baseline and zero-out simulations (ppb), showing the population- weighted average for each region of the average 1-hr daily maximum ozone for the consecutive 6-month period with the highest average.
Table S4 – $PM_{2.5}$ concentrations for baseline and zero-out simulations ($\mu g/m^3$), showing the population- weighted average for each region of the annual average concentrations.
Table S5 – Concentrations of seven $PM_{2.5}$ species in the baseline simulation ($\mu g/m^3$), showing the population- weighted average for each region of the annual average concentrations.
Table S6 – Regional and global total population, exposed population (adults age 25 and older), and average cause-specific mortality rates (for the exposed population).
Table S7 – Premature ozone-related respiratory mortality in ten world regions, and contributions from different sectors (deaths in 2005), showing the mean and 95% CI from 1000 Monte Carlo simulations. All numbers are rounded to three significant digits. Regional mean values correspond to the values shown in Figure 3 of the main paper.
Table S8 – Premature ozone-related respiratory mortality in ten world regions, and contributions from different sectors (deaths in 2005), showing the deterministic mean estimates. All numbers are rounded to three significant digits.
Table S9 – Premature ozone-related respiratory mortality per million people in total population in ten world regions, and contributions from different sectors (deaths per million people in 2005), showing the mean from 1000 Monte Carlo simulations. All numbers are rounded to the nearest unit.
Table S10 – Premature $PM_{2.5}$-related mortality in ten world regions (IHD+Stroke+COPD+LC), and contributions from different sectors (deaths in 2005), showing the mean and 95% CI from 1000 Monte Carlo simulations. All numbers are rounded to three significant digits. Regional mean values correspond to the values shown in Figure 5 of the main paper.
Table S11 – Premature $PM_{2.5}$-related mortality (IHD+Stroke+COPD+LC) in ten world regions, and contributions from different sectors (deaths in 2005), showing the deterministic mean estimates. All numbers are rounded to three significant digits.

Table S12 – Premature $PM_{2.5}$ -related mortality (IHD+Stroke+COPD+LC) per million people in total population in ten world regions, and contributions from different sectors (deaths per million people in 2005), showing the mean from 1000 Monte Carlo simulations. All numbers are rounded to the nearest unit.

Table S13 – Premature ozone and $PM_{2.5}$-related mortality in ten world regions for the contribution of all anthropogenic emissions at 2.5°x1.9° horizontal resolution (deaths in 2005), showing the deterministic mean estimates. All numbers are rounded to three significant digits.

Table S14 – Premature ozone and $PM_{2.5}$-related mortality in ten world regions for the contribution of all anthropogenic emissions for the simulations at 0.67°x0.5° regridded to 2.5°x1.9° horizontal resolution (deaths in 2005), showing the deterministic mean estimates. All numbers are rounded to three significant digits.

Figure S1 – Comparison of simulated monthly mean surface ozone concentrations (2005) with CASTNet monitored concentrations (2005) for eight US regions, showing modeled regional mean (red), CASTNET regional mean (black) and individual monitoring locations (grey). An overall model bias of 7.2 ppbv is calculated across all stations.

Figure S2 – Comparison of simulated monthly mean surface ozone concentrations (2005) with EMEP monitored concentrations (2005) for six European regions, showing modeled regional mean (red), EMEP regional mean (black) and individual monitoring locations (grey). An overall model bias of 2.3 ppbv is calculated across all stations.

Figure S3 – Comparison of the simulated annual average (2005) surface SO_4 concentrations (μg m^{-3}) with annual average (2005) observations from the IMPROVE(A) and the EMEP (B) surface monitoring networks for the US and Europe, respectively. The left panels show modeled versus observed concentrations (μg m^{-3}) with the dashed 1:2 and 2:1 lines representing agreement within a factor of 2. The right panels show a map of [(modeled-observed)/observed] values.

Figure S4 – Comparison of the simulated annual average (2005) surface NH_4NO_3 concentrations (μg m^{-3}) with annual average (2005) observations from the IMPROVE (A) and the EMEP (B) surface monitoring networks for the US and Europe, respectively. The left panels show modeled versus observed concentrations (μg m^{-3}) with the dashed 1:2 and 2:1 lines representing

agreement within a factor of 2. The right panels show a map of [(modeled-observed)/observed] values.

Figure S5 – Comparison of the simulated annual average (2005) surface EC concentrations (µg m^{-3}) with annual average (2005) observations from the IMPROVE surface monitoring networks, US. The left panels show modeled versus observed concentrations (µg m^{-3}) with the dashed 1:2 and 2:1 lines representing agreement within a factor of 2. The right panels show a map of [(modeled-observed)/observed] values.

Figure S6 – Comparison of the simulated annual average (2005) surface OC concentrations (µg m^{-3}) with annual average (2005) observations from the IMPROVE surface monitoring networks, US. The left panels show modeled versus observed concentrations (µg m^{-3}) with the dashed 1:2 and 2:1 lines representing agreement within a factor of 2. The right panels show a map of [(modeled-observed)/observed] values.

Figure S7 – Ten World Regions.

Figure S8 – Ozone concentrations in the baseline simulation (ppb), showing the average 1-hr daily maximum ozone for the consecutive 6-month period with the highest average in each cell.

Figure S9 – Difference in ozone concentrations between the baseline and zeroed-out simulations (ppb), using the average 1-hr daily maximum ozone for the consecutive 6-month period with the highest average in each cell.

Figure S10 – $PM_{2.5}$ concentrations in the baseline simulation (µg/m^3), showing the annual average concentrations in each cell.

Figure S11 – Fraction of selected $PM_{2.5}$ species (A – D) in total $PM_{2.5}$ concentrations in the baseline simulation.

Figure S12 – Difference in $PM_{2.5}$ concentrations between the baseline and zeroed-out simulations (A – F) (µg/m^3), using the annual average concentrations in each cell.

1. Input emissions

Input anthropogenic and biomass burning emissions were processed from datasets at 0.5° x 0.5° horizontal resolution prepared for the IPCC's Fifth Assessment Report and obtained through the GEIA-ACCENT Emission Data Portal[1], for the IPCC AR5 Representative Concentration Pathway 8.5 (RCP8.5) global emissions inventory (Riahi et al. 2011) for 2005.

Anthropogenic emissions were processed as described by Fry et al. (2013), including speciation of volatile organic compound (VOC) species to MOZART-4 VOC categories, adding monthly temporal variation to all emissions species from anthropogenic sources, and regridding to 0.67°x0.5° horizontal resolution. We applied 1.15 and 1.4 factors to black and organic carbon species, respectively, to adjust for the conversion of emissions from PM_1 to $PM_{2.5}$ in the Bond inventory (Bond et al. 2004; Cooke et al. 1999) used in the emissions dataset, following Liu et al. (2009), Anenberg et al. (2011) and West et al. (2013).

The baseline 2005 simulation was run with emissions from the 10 anthropogenic and 2 biomass burning emissions sectors included in the global emissions dataset (Tables S1 and S2).

2. MOZART-4 performance evaluation

MOZART-4 performance was evaluated by comparing surface concentrations of ozone and $PM_{2.5}$ species (EC, OC, SO_4, NH_4NO_3) with 2005 surface air quality observations from the Clean Air Status and Trends Network (CASTNet) for the US (ozone), the European Monitoring and Evaluation Programme (EMEP) for Europe (ozone and $PM_{2.5}$ species) and the Interagency

[1] http://accent.aero.jussieu.fr/database_table_inventories.php

Monitoring of Protected Visual Environments (IMPROVE) for remote locations in the U.S. ($PM_{2.5}$ species).

Figures S1 and S2 show comparisons of modeled surface ozone with observations from CASTNET and EMEP, respectively. For ozone, overall mean bias is 7.2 ppb for CASTNet and 2.3 ppb for EMEP. Throughout the year, the model shows higher biases in the US North West and Florida regions and, during the Summer, the highest biases are in the Great Lakes, North East and South East regions (regional average bias less than 25 ppb). In Europe, bias is lower than in the US.

Figures S3 to S6 show comparisons of modeled surface concentrations of $PM_{2.5}$ species with observations from IMPROVE and EMEP. For $PM_{2.5}$ species, the model shows good agreement with observations. Simulated annual average surface sulfate concentrations are mostly within a factor of two of observations, though with a tendency to underestimate. Simulated annual average ammonium nitrate concentrations are generally overestimated. EC and OC simulated annual average concentrations show very good agreement with observations in the US.

3. Simulation with zeroed-out anthropogenic emissions

For this simulation we zeroed-out emissions from all anthropogenic sectors (i.e. Energy, Industry, Land Transportation, Shipping, Aviation, Residential & Commercial, Solvents, Agriculture, Agricultural Waste Burning, Waste). Also, we set total soil NOx emissions to a preindustrial value (3.6 Tg N yr^{-1}, estimated by Yienger and Levy, 1995), following Horowitz (2006), and we fixed methane concentrations at 722 ppb (Myhre et al. 2013).

For biomass burning emissions, we took into account that climate was likely the main driver of global fire activity up to the early 18th century. In contrast, anthropogenic influence plays a major role afterwards; fire ignition is the greatest contributor to the increase in global fire activity up to the end of the 19th century and fire suppression is responsible for a considerable decrease in global fire activity in the 20th century (Marlon et al. 2008; Pechony and Shindell 2010; Power et al. 2012 and references therein). Considering that present-day savannah fires are mostly ignited by humans (Andrae, 1991; Schultz et al. 2008), that conditions in closed tropical forests are usually not conducive to biomass burning (Krawchuk et al. 2009) and that human-ignited fires related to tropical deforestation have greatly increased in recent decades (Mieville et al. 2010), we assumed natural emissions from savannah and tropical forest fires to be 10% of present-day emissions. We took into account that fire emissions likely increased substantially between 1750 and the late 1800s due to anthropogenic fire ignitions (Pechony and Shindell 2010; Power et al. 2012 and references therein), so a 50% assumption (Moulliot et al. 2006) would not be applicable. For forest fires at extratropical latitudes (greater than 30° N and 20° S), we assumed natural emissions to be 90% of present-day emissions to account for the reduced effect of anthropogenic ignition in these regions (Andrae, 1991; Eliseev et al. 2014), although others suggest that a lower percentage may be appropriate in the boreal Eurasian forests (Mollicone et al. 2006).

4. Ozone and $PM_{2.5}$ surface concentrations

All averaged concentrations mentioned in this section are population-weighted averages, unless otherwise stated. All regional results refer to the regions shown in Figure S7.

4.1 Ozone

Modeled hourly ozone concentrations were processed to estimate the average 1-hr daily maximum concentration for the consecutive 6-month period with the highest average, in each grid cell.

Ozone concentrations in the baseline simulation were 56.0 ppb globally, ranging from 39.9 to 64.1 ppb across ten world regions, with the highest values in East Asia, India and US (Table S3 and Figure S8). When all anthropogenic emissions were zeroed-out, global concentrations decreased to 22.2 ppb (40% of baseline) and regional concentrations ranged from 17.0 to 24.3 ppb, reflecting a much lower spatial variability in surface ozone (Table S3). Considering the results of the simulations with the different sectors zeroed-out (Table S3 and Figure S9), All Transportation had the highest contribution to surface ozone, globally (14%) and across all regions, with Land Transportation accounting for close to 70% of the global contribution from All Transportation. Land Transportation had the greatest individual contribution in most regions, except Africa (Residential & Commercial) and Southeast Asia and Australia (Energy). In East Asia, the contribution of Industry was very close to Land Transportation.

4.2 $PM_{2.5}$

Annual average $PM_{2.5}$ concentrations were estimated as a sum of individual model output species:

PM2.5 = CB1+CB2 + (OC1*1.3+OC2*1.7) + SO4+ NO3 + SOA +

+ 0.2*(DUST1+DUST2+DUST3) + (SA1+SA2+SA3)

CB1 and CB2 are black carbon species. OC1 and OC2 are primary organic carbon species. The 1.3 and 1.7 factors applied to OC1 and OC2 account for species other than carbon in organic aerosol. SO4 is sulfate (estimated as ammonium sulfate). NO3 is nitrate (estimated as ammonium nitrate). SOA is secondary organic aerosol. DUST1, DUST2 and DUST3 and SA1, SA2 and SA3 are the size fractions of dust and sea salt relevant for $PM_{2.5}$ (size bins 1-3). Since modeled windblown dust is highly uncertain and caused $PM_{2.5}$ model estimates to be unrealistically large in arid regions, dust was adjusted with a 0.2 factor so that global $PM_{2.5}$ surface concentrations roughly agree with those estimated by Brauer et al. (2012), as had been done by West et al. (2013).

$PM_{2.5}$ concentrations in the baseline simulation were 22.0 $\mu g/m^3$ globally, ranging from 5.0 to 34.2 $\mu g/m^3$ regionally, with the highest values in East Asia, India and Middle East (Table S4 and Figure S10). Considering different species, global black carbon averages 1.4 $\mu g/m^3$, primary organic aerosol (POA) averages 6.5 $\mu g/m^3$ and dust averages 5.8 $\mu g/m^3$, while SOA averages 0.1$\mu g/m^3$ and secondary inorganic aerosol (SIA = NO3+SO4) averages 4.2 $\mu g/m^3$ (Table S5 and Figure S11). Globally, POA corresponds to the greatest fraction of total $PM_{2.5}$ concentration (29%), followed closely by dust (26%); however, in some regions other species account for the greatest fractions, such as sulfate in North America (31%), nitrate in Europe (43%) and East Asia (33%), and dust in Africa (65%) and Middle East (72%).

When all anthropogenic emissions were zeroed-out, global concentrations decreased to 6.6 $\mu g/m^3$ (30% of baseline) and regional concentrations ranged from 1.4 to 20.6 $\mu g/m^3$ with the highest value in the Middle East (Table S4). Also, the reduction was the greatest in East Asia

(less 91%) and the least in the Middle East (less 26%) and Africa (less 30%), due to the fraction of dust in these latter regions.

Considering the results of the simulations with the different sectors zeroed-out (Table S4 and Figure S12), Residential & Commercial had the highest contribution to surface $PM_{2.5}$, globally (28%) and in several regions. Land Transportation had the greatest individual contribution in North America and Europe.

5. Population and Baseline Mortality Rates

Table S6 shows the global and regional total population, exposed population (adults aged 25 and older), and cause-specific baseline mortality rates obtained from the gridded values used in the health impact assessment.

6. Additional mortality results

Tables S7 and S10 show present-day global and regional burdens of anthropogenic ozone and $PM_{2.5}$-related mortality.

We also obtained deterministic estimates for mortality, considering the mean values reported for each of the variables (see Tables S8 and S11). For anthropogenic ozone mortality, the global mean from the Monte Carlo simulations is 0.1% greater than the deterministic estimate (492,000 deaths/year), with less than ±1% differences for most regions, except Middle East (2%) and Africa (5%). For anthropogenic $PM_{2.5}$ mortality, there is a larger difference (-5%) between the global mean from the Monte Carlo simulation and the deterministic estimate (2.36

million deaths/year), reflecting large differences at the regional level in some regions (e.g. -15% in India, -4% in East Asia, -6% in Europe, 19% in North America).

Tables S9 and S12 show ozone- and $PM_{2.5}$-related total mortality rates per sector, considering total population, per region and globally. India and East Asia have 113 deaths per million people per year due to exposure to anthropogenic ozone, compared to 69 deaths per million people in North America and lower rates in all other regions (Table S9). The greatest sectoral global impact is due to Land Transportation, which contributes 12 deaths per million people per year.

FSU has the highest anthropogenic $PM_{2.5}$-related mortality rate (714 deaths/year per million people), closely followed by East Asia (683 deaths/year per million people), while Europe and India have 457 and 268 deaths/year per million people, respectively (Table S12). The greatest global sectoral impact is due to Residential & Commercial, which contributes 97 deaths per million people per year.

7 Sensitivity Analysis (additional information)

7.1 Fine vs. coarse resolution

Using the output from simulations at 2.5°x1.9° horizontal resolution, we estimate a present-day global burden of anthropogenic ozone-related mortality of 480,000 deaths/year, a slight negative bias of 2% relative to the deterministic estimate at finer resolution (Table S13); this negative bias was greater in some regions (e.g. South America, -9%; Southeast Asia, -8%; Africa, -6%; Australia, -12%). For anthropogenic $PM_{2.5}$ mortality, we estimate 2.7 million deaths/year, which corresponds to a global positive bias of 16% for the coarse resolution

estimate, resulting from considerably different regional bias, including much larger positive biases in North America (52%), Europe (34%) and FSU (39%), smaller positive biases in India (4%), East Asia (11%) and Southeast Asia (11%), and a 15% negative bias in South America (Table S13).

If we regrid the output from simulations at 0.67°x0.5° horizontal resolution to 2.5°x1.9° horizontal resolution, we estimate a present-day global burden of anthropogenic ozone-related mortality of 479,000 deaths/year, a slight negative bias of 3% relative to the deterministic estimate at finer resolution, while for anthropogenic $PM_{2.5}$ mortality we estimate a burden of 2.2 million deaths /year, corresponding to a negative bias of 8% (Table S14).

7.2 Low-concentration threshold for ozone

By applying a low-concentration threshold of 33.3ppb to the ozone mortality calculation, we estimate a 27.4% decrease in the global mortality burden, since ozone concentrations for the simulation with all anthropogenic emissions zeroed-out are below the threshold, except in a few very small areas in Africa. The low-concentration threshold has a negligible effect on the contributions of each sector (less than 2%) since ozone concentrations with zeroed-out sectors are lower than the threshold only in a few populated areas, mostly in South America and Australia.

References

Andreae MO. 1991. Biomass burning: Its history, use and distribution and its impact on environmental quality and global climate. In *Global Biomass Burning: Atmospheric, Climatic and Biospheric Implications* (J.S. Levine ed.), pp. 3–21, MIT Press, Cambridge, Mass.

Anenberg SC, Talgo K, Arunachalam S, Dolwick P, Jang C, West JJ. 2011. Impacts of global, regional, and sectoral black carbon emission reductions on surface air quality and human mortality. Atmos Chem Phys 11:7253–7267; doi:10.5194/acp-11-7253-2011.

Bond TC, Streets DG, Yarber KF, Nelson SM, Woo JH, Klimont Z. 2004. A technology-based global inventory of black and organic carbon emissions from combustion. J Geophys Res D Atmos 109:1–43; doi:10.1029/2003JD003697.

Brauer M, Amann M, Burnett RT, Cohen A, Dentener F, Ezzati M, et al. 2012. Exposure assessment for estimation of the global burden of disease attributable to outdoor air pollution. Environ Sci Technol 46:652–60; doi:10.1021/es2025752.

Bright EA, Coleman PR, Rose AN, Urban ML. 2012. LandScan 2011. Oak Ridge National Laboratory SE, Oak Ridge, TN.

Burnett RT, Arden Pope C, Ezzati M, Olives C, Lim SS, Mehta S, et al. 2014. An integrated risk function for estimating the global burden of disease attributable to ambient fine particulate matter exposure. Environ Health Perspect 122:397–403; doi:10.1289/ehp.1307049.

Cooke WF, Liousse C, Cachier H, Feichter J. 1999. Construction of a 1° x 1° fossil fuel emission data set for carbonaceous aerosol and implementation and radiative impact in the ECHAM4 model. J Geophys Res 104: 22137–22162.

Eliseev A V, Mokhov II, Chernokulsky A V. 2014. An ensemble approach to simulate CO 2 emissions from natural fires. Biogeosciences 11:3205–3223; doi:10.5194/bg-11-3205-2014.

Fry MM, Schwarzkopf MD, Adelman Z, Naik V, Collins WJ, West JJ. 2013. Net radiative forcing and air quality responses to regional CO emission reductions. Atmos Chem Phys 13:5381–5399; doi:10.5194/acp-13-5381-2013.

Horowitz LW. 2006. Past , present , and future concentrations of tropospheric ozone and aerosols : Methodology , ozone evaluation , and sensitivity to aerosol wet removal. J Geophys Res 111:D22211; doi:10.1029/2005JD006937.

Institute for Health Metrics and Evaluation (IHME). 2013. Global Burden of Disease Study 2010 (GBD 2010) Results by Cause 1990-2010 - Country Level. Seattle, United States.

Krawchuk MA, Moritz MA, Parisien M-A, Van Dorn J, Hayhoe K. 2009. Global Pyrogeography : the Current and Future Distribution of Wildfire. PLoS One 4:e5102; doi:10.1371/journal.pone.0005102.

Krewski D, Jerrett M, Burnett RT, Ma R, Hughes E, Shi Y, et al. 2009. Extended Follow-Up and Spatial Analysis of the American Cancer Society Study Linking Particulate Air Pollution and Mortality. Respir Rep Heal Eff Inst 140: 5–114.

Lamarque JF, Bond TC, Eyring V, Granier C, Heil A, Klimont Z, et al. 2010. Historical (1850-2000) gridded anthropogenic and biomass burning emissions of reactive gases and aerosols: Methodology and application. Atmos Chem Phys 10:7017–7039; doi:10.5194/acp-10-7017-2010.

Lamarque JF, Shindell DT, Josse B, Young PJ, Cionni I, Eyring V, et al. 2013. The atmospheric chemistry and climate model intercomparison Project (ACCMIP): Overview and description of models, simulations and climate diagnostics. Geosci Model Dev 6:179–206; doi:10.5194/gmd-6-179-2013.

Liu J, Mauzerall DL, Horowitz LW, Ginoux P, Fiore AM. 2009. Evaluating inter-continental transport of fine aerosols: (1) Methodology, global aerosol distribution and optical depth. Atmos Environ 43:4327–4338; doi:10.1016/j.atmosenv.2009.03.054.

Marlon JR, Bartlein PJ, Carcaillet C, Gavin DG, Harrison SP, Higuera PE, et al. 2008. Climate and human influences on global biomass burning over the past two millennia. Nat Geosci 1:697–702; doi:10.1038/ngeo468

Mieville A, Granier C, Liousse C, Guillaume B, Mouillot F, Lamarque JF, et al. 2010. Emissions of gases and particles from biomass burning during the 20th century using satellite data and an historical reconstruction. Atmos Environ 44:1469–1477; doi:10.1016/j.atmosenv.2010.01.011.

Mollicone D, Eva HD, Achard F. 2006. Human role in Russian wild fires. Nature 440:436–437; doi:10.1038/440435a.

Mouillot F, Narasimha A, Balkanski Y, Lamarque J-F, Field CB. 2006. Global carbon emissions from biomass burning in the 20th century. Geophys Res Lett 33:2–5; doi:10.1029/2005GL024707.

Myhre G, Shindell D, Bréon F-M, Collins W, Fuglestvedt J, Huang J, et al. 2013. Anthropogenic and Natural Radiative Forcing. In Climate Change 2013: The Physical Science Basis. Contribution of Working Group I to the Fifth Assessment Report of the Intergovernmental Panel on Climate Change (Stocker TF, Qin D, Plattner G-K, Tignor M, Allen SK, Boschung J, et al. eds.), pp. 659–740, Cambridge University Press, Cambridge, United Kingdom and New York, NY, USA.

Pechony O, Shindell DT. 2010. Driving forces of global wildfires over the past millennium and the forthcoming century. Proc Natl Acad Sci USA 107:19167–19170; doi:10.1073/pnas.1003669107.

Power MJ, Mayle FE, Bartlein PJ, Marlon JR, Anderson RS, Behling H, et al. 2012. Climatic control of the biomass-burning decline in the Americas after AD 1500. The Holocene 23:3; doi:10.1177/0959683612450196.

Riahi K, Rao S, Krey V, Cho CH, Chirkov V, Fischer G, et al. 2011 RCP 8.5-A scenario of comparatively high greenhouse gas emissions. Clim Ch 109: 33-57; doi:10.1007/s10584-011-0149-y.

Schultz MG, Heil A, Hoelzemann JJ, Spessa A, Thonicke K, Goldammer JG, et al. 2008. Global wildland fire emissions from 1960 to 2000. Global Biogeochem Cycles 22:1–17; doi:10.1029/2007GB003031.

Silva RA, West JJ, Zhang Y, Anenberg SC, Lamarque J-F, Shindell DT, et al. 2013. Global premature mortality due to anthropogenic outdoor air pollution and the contribution of past climate change. Environ Res Lett 8:034005; doi:10.1088/1748-9326/8/3/034005.

West JJ, Smith SJ, Silva RA, Naik V, Zhang Y, Adelman Z, et al. 2013. Co-benefits of Global Greenhouse Gas Mitigation for Future Air Quality and Human Health. Nat Clim Chang 3:885–889; doi:10.1038/NCLIMATE2009.

Yienger JJ and Levy, H. 1995. Empirical model of global soil-biogenic NOx emissions. J Geophys Res 100: 11447–11464.

Table S1 – Emissions source sectors included in the RCP8.5 global emissions inventory.

Sector code	Short name	Definition (based on IPCC/CRF source/sink sectors)
Anthropogenic Emissions		
Ene	Energy	Energy production and distribution: Public electricity and heat production, petroleum refining, manufacture of solid fuels and other energy industries. Fugitive emissions from solid fuels, oil and gas.
Ind	Industry	Industrial processes and combustion: Manufacturing industries and construction, inc. production of metals, chemicals, pulp /paper / print, food processing/beverages/tobacco, and other industries.
tra*	Land Transportation	Land transport: Road transportation (cars, light duty trucks, heavy duty trucks and buses, motorcycles, evaporative emissions from vehicles), railways, and other transportation (pipeline transport, off-road).
shp*	Shipping	Maritime transport: International shipping
aircraft*	Aviation	Civil Aviation
Dom	Residential & Commercial	Residential and commercial combustion**
Slv	Solvents	Solvent production and use
Agr	Agriculture	Agriculture (animals, rice, soil): Enteric fermentation, manure management, rice cultivation, soil emissions, other
Awb	Agricultural Waste Burning	Agriculture (waste burning on fields): Field burning of agricultural residues
Wst	Waste	Waste Treatment and Disposal: Solid waste disposal on land, wastewater handling, waste incineration
Biomass Burning Emissions		
Lcf	Forest burning	Forest fires
Sav	Grassland burning	Savanna burning, grassland fires

* All Transportation includes these three sectors.

** According to Lamarque et al. (2010), includes emissions from fuelwood burning and charcoal production.

Table S2 – Global emissions per sector for six pollutants (Tg/yr) in the RCP 8.5 Global Emissions Inventory, 2005, and the total emissions for all 12 sectors and for the sectors modeled in zero-out simulations (and percentage of the modeled sectors in total emissions in parenthesis).

	CO	NO	NMVOC	SO_2	BC	OC
Ene	21.1	17.1	29.1	57.7	0.05	0.3
Dom	261.3	6.2	38.6	8.8	2.1	8.2
Ind	114.3	11.2	9.0	26.9	1.6	2.3
Tra	161.8	20.5	28.5	3.4	1.2	1.3
Wst	4.1	0.2	1.5	0.05	0.04	0.05
Agr	0.01	1.5	0.8	-	-	-
Slv	1.0		22.7	-	-	-
Awb	19.9	0.4	2.7	0.2	0.1	0.7
Shp	1.3	12.3	3.1	13.0	0.1	0.1
Aircraft	-	0.8	-	-	0.003	-
Lcf	229.3	3.9	41.7	2.2	1.1	12.0
Sav	222.3	7.5	35.1	1.6	1.5	10.9
Total	1036	81.7	212.9	113.9	7.9	35.9
modeled sectors (% of total)	559.8	68.1	108.4	109.9	5.2	12.2
(ene+dom+ind+tra+shp+aircraft)	(54%)	(83%)	(51%)	(96%)	(65%)	(34%)

Table S3 – Ozone concentrations for baseline and zero-out simulations (ppb), showing the population- weighted average for each region of the average 1-hr daily maximum ozone for the consecutive 6-month period with the highest average.

	**Baseline**	**Residential & Comm.**	**Energy**	**Industry**	**Land Transp.**	**All Transp.**	**All Anthr.**
**NA**	59.8	58.1	56.0	57.0	51.4	47.5	20.3
**SA**	39.9	39.0	38.4	37.4	34.6	33.0	18.6
**Europe**	53.5	52.1	51.4	51.6	47.0	42.1	20.9
**FSU**	48.4	47.4	45.2	46.7	43.2	40.8	21.8
**Africa**	45.9	42.0	44.7	45.3	44.0	42.4	23.4
**India**	60.5	55.2	54.8	58.6	54.8	53.0	24.3
**East Asia**	64.1	60.3	59.6	58.8	58.7	56.7	23.8
**SE Asia**	52.1	49.5	46.0	49.7	46.6	43.8	17.0
**Australia**	40.0	39.1	36.1	36.1	37.7	34.9	17.3
**ME**	57.4	55.3	53.3	55.1	49.5	45.8	22.7
**Global**	**56.0**	**52.8**	**52.0**	**53.3**	**50.5**	**48.0**	**22.2**

Table S4 – $PM_{2.5}$ concentrations for baseline and zero-out simulations (μg/m^3), showing the population- weighted average for each region of the annual average concentrations.

	Baseline	Residential & Comm.	Energy	Industry	Land Transp.	All Transp.	All Anthr.
NA	8.5	7.6	7.1	7.5	6.4	6.2	1.4
SA	6.7	5.9	6.4	5.0	5.7	5.6	2.0
Europe	13.9	11.1	12.0	12.5	10.9	9.9	2.7
FSU	12.1	8.4	10.8	11.3	11.2	11.1	3.6
Africa	16.8	14.9	16.4	16.5	16.6	16.5	11.7
India	28.5	16.3	24.3	25.4	26.6	26.5	8.5
East Asia	34.2	23.3	28.3	25.3	32.0	31.5	3.1
SE Asia	12.0	8.1	10.6	10.4	11.1	10.9	1.9
Australia	5.0	4.7	4.6	4.6	4.7	4.6	3.1
ME	27.8	25.7	26.1	26.5	26.1	25.8	20.6
Global	22.0	15.7	19.4	18.7	20.4	20.1	6.6

Table S5 – Concentrations of seven $PM_{2.5}$ species in the baseline simulation (μg/m^3), showing the population- weighted average for each region of the annual average concentrations.

	BC	POA	Sulfate	SOA	Nitrate	Dust	Sea Salt
NA	0.6	2.2	2.6	0.1	2.1	0.5	0.4
SA	0.6	2.9	1.5	0.1	0.2	0.8	0.6
Europe	0.8	2.4	2.4	0.0	5.9	1.5	0.8
FSU	0.5	4.1	1.6	0.0	2.7	2.9	0.2
Africa	0.5	3.9	0.9	0.1	0.2	11.0	0.3
India	1.8	10.9	3.6	0.1	3.8	8.0	0.3
East Asia	3.0	10.0	7.1	0.1	11.3	2.4	0.3
SE Asia	0.8	5.9	3.4	0.2	0.5	0.7	0.5
Australia	0.3	1.0	0.9	0.1	0.2	0.9	1.6
ME	0.6	2.5	2.7	0.0	1.5	19.9	0.5
Global	1.4	6.5	3.6	0.1	4.3	5.8	0.4

Table S6 – Regional and global total population, exposed population (adults age 25 and older), and average cause-specific mortality rates (for the exposed population).

	Total Population (millions)	Population 25+ (millions)	Mortality Rates per 100,000 (for population 25 and over)				
			IHD	Stroke	COPD	LC	Resp
NA	538.7	330.5	234.0	84.6	62.2	61.5	75.4
SA	404.1	225.7	141.8	103.9	40.9	23.0	58.5
Europe	516.7	375.0	285.3	178.4	54.7	72.1	73.5
FSU	293.6	196.0	684.5	362.7	42.9	49.1	52.3
Africa	885.6	315.1	65.5	98.3	18.7	6.3	37.1
India	1,460.2	692.3	169.9	95.1	137.8	14.2	178.2
East Asia	1,553.2	1,036.5	119.1	207.2	104.7	62.8	117.5
SE Asia	644.9	351.4	118.2	187.8	42.2	30.6	71.3
Australia	28.0	18.5	215.9	97.4	49.0	54.0	62.4
ME	621.1	297.7	190.3	120.3	29.4	21.2	51.2
Global	6,946.3	3,838.8	186.1	157.5	76.3	40.9	97.5

Sources:
Population - Oak Ridge National Laboratory's Landscan 2011 Global Population Dataset (Bright et al. 2012).
Baseline mortality rates - Cause-specific baseline mortality rates for 187 countries (IHME 2013)

Table S7 – Premature ozone-related respiratory mortality in ten world regions, and contributions from different sectors (deaths in 2005), showing the mean and 95% CI from 1000 Monte Carlo simulations. All numbers are rounded to three significant digits. Regional mean values correspond to the values shown in Figure 3 of the main paper.

Region	All Anthropogenic	All Transportation	Land Transportation	Energy	Industry	Residential & Commercial
North America	37,100<br>(8,380 – 74,800)	12,100<br>(2,840 - 25,600)	8,390<br>(1,790 - 18,400)	3,880<br>(880 - 8,390)	3,020<br>(594 – 6,360)	1,440<br>(347 – 3,040)
South America	10,300<br>(302 - 23,600)	3,450<br>(292 - 7,950)	2,650<br>(212 – 6,420)	787<br>(38 - 1,890)	1,270<br>(0 - 2,990)	416<br>(30 – 958)
Europe	32,800<br>(9,000 - 64,700)	12,100<br>(3,2030 - 24,700)	6,620<br>(1,840 - 14,100)	2,170<br>(610 - 4,520)	2,030<br>(498 – 4,100)	1,450<br>(360 – 3,000)
F. Soviet Union	10,300<br>(3,230 - 19,700)	3,090<br>(9470 - 6,070)	2,040<br>(645 - 4,120)	1,230<br>(370 - 2,420)	684<br>(1204 - 1,350)	402<br>(120 – 790)
Africa	10,200<br>(3,050 - 22,900)	1,700<br>(494 - 3,930)	907<br>(267 - 2,040)	578<br>(151 - 1,310)	303<br>(79 – 699)	1,490<br>(431 - 3,280)
India	165,000<br>(40,200 - 343,000)	35,900<br>(8,200 – 78,500)	27,100<br>(5,560 - 59,900)	27,500<br>(6,380 - 60,500)	9,160<br>(1,840 – 20,000)	26,000<br>(5,770 – 57,300)
East Asia	175,000<br>(34,100 - 358,000)	32,600<br>(6,000 - 70,900)	23,500<br>(4,320 – 53,100)	20,900<br>(4,0670 - 46,600)	25,200<br>(4,320 - 53,800)	18,500<br>(3,600 - 39,600)
Southeast Asia	32,400<br>(8,000 - 66,900)	7,550<br>(1,780 - 16,200)	5,080<br>(980 - 11,300)	5,720<br>(920 - 13,100)	2,340<br>(426 – 5,300)	2,700<br>(659 - 5,790)
Australia	979<br>(136 - 2,130)	232<br>(35 – 520)	103<br>(15 – 241)	180<br>(25 – 417)	176<br>(24 – 388)	38<br>(5 – 86)
Middle East	19,300<br>(6,270 - 39,300)	6,690<br>(2,110 - 13,800)	4,570<br>(1,420 - 9,460)	2,220<br>(634 - 4,750)	1,380<br>(415 - 2,940)	1,350<br>(390 - 2,920)
Global	493,000<br>(122,000 – 989,000)	115,000<br>(27,800 - 244,000)	80,900<br>(17,400 – 180,000)	65,200<br>(14,500 – 143,000)	45,600<br>(8,700 – 96,800)	53,700<br>(12,300 – 116,000)

Table S8 – Premature ozone-related respiratory mortality in ten world regions, and contributions from different sectors (deaths in 2005), showing the deterministic mean estimates. All numbers are rounded to three significant digits.

	All Anthr.	All Transp.	Land Transp.	Energy	Industry	Resid. & Comm.
NA	37,400	12,000	8,500	3,960	3,020	1,430
SA	10,400	3,430	2,680	803	1,270	415
Europe	32,800	12,000	6,670	2,200	2,020	1,440
FSU	10,200	3,040	2,050	1,240	678	400
Africa	9,670	1,600	875	566	289	1,430
India	163,000	35,200	27,000	27,700	9,020	25,700
East Asia	176,000	32,300	23,800	21,400	25,300	18,500
SE Asia	32,200	7,400	5,080	5,790	2,310	2,680
Australia	979	229	104	183	175	38
ME	18,900	6,490	4,510	2,200	1,340	1,330
Global	492,000	114,000	81,300	66,000	45,400	53,400

Table S9 – Premature ozone-related respiratory mortality per million people in total population in ten world regions, and contributions from different sectors (deaths per million people in 2005), showing the mean from 1000 Monte Carlo simulations. All numbers are rounded to the nearest unit.

	All Anthr.	All Transp.	Land Transp.	Energy	Industry	Resid. & Comm.
NA	69	23	16	7	6	3
SA	26	9	7	2	3	1
Europe	63	23	13	4	4	3
FSU	35	11	7	4	2	1
Africa	11	2	1	1	0	2
India	113	25	19	19	6	18
East Asia	113	21	15	13	16	12
SE Asia	50	12	8	9	4	4
Australia	35	8	4	6	6	1
ME	31	11	7	4	2	2
Global	71	17	12	9	7	8

Table S10 – Premature $PM_{2.5}$-related mortality in ten world regions (IHD+Stroke+COPD+LC), and contributions from different sectors (deaths in 2005), showing the mean and 95% CI from 1000 Monte Carlo simulations. All numbers are rounded to three significant digits. Regional mean values correspond to the values shown in Figure 5 of the main paper.

Region	All Anthropogenic	All Transportation	Land Transportation	Energy	Industry	Residential & Commercial
North America	91,100 (6,200 - 223,000)	33,600 (5,180 – 60,600)	29,200 (4,540 - 51,700)	20,700 (1,290 - 40,600)	13,200 (1,490 - 23,500)	11,800 (1,820 - 20,400)
South America	31,000 (1,220 - 75,100)	8,190 (1,020 – 13,900)	7,600 (1,010 - 12,800)	1,990 (187 - 3,760)	13,100 (1,090 - 23,100)	5,800 (965 - 10,100)
Europe	236,000 (59,900 - 382,000)	76,700 (27,500 – 112,000)	56,200 (22,600 - 80,900)	38,300 (21,800 - 53,500)	25,600 (13,000 - 36,100)	64,000 (39,300 - 88,100)
F. Soviet Union	210,000 (48,400 - 334,000)	30,000 (15,000 – 38,300)	26,400 (13,600 - 33,600)	37,100 (16,100 - 47,400)	21,100 (11,800 - 26,600)	113,000 (41,300 - 144,000)
Africa	28,600 (3,530 - 57,700)	1,940 (560 – 3,170)	1,540 (505 - 2,480)	2,760 (752 - 4,690)	2,030 (466 - 3,470)	12,000 (2,250 - 23,300)
India	392,000 (129,000 - 590,000)	21,800 (11,700 – 31,600)	19,900 (11,100 -28,800)	39,200 (18,900 - 57,100)	36,400 (18,900 - 52,500)	173,000 (88,000 - 253,000)
East Asia	1,060,000 (696,000 - 1,440,000)	61,000 (44,300 – 78,800)	48,000 (34,700 - 62,500)	117,000 (84,500 - 156,000)	178,000 (127,000 - 239,000)	223,000 (158,000 - 299,000)
Southeast Asia	120,000 (30,900 - 215,000)	14,800 (5,650 – 22,300)	12,400 (4,990 - 18,000)	20,300 (8,160 - 29,700)	24,600 (9,080 - 36,000)	59,900 (24,200 - 90,900)
Australia	922 (0 - 3,820)	309 (0 – 1,020)	201 (0 – 638)	292 (0 – 920)	333 (0 - 1,070)	223 (0 – 681)
Middle East	59,700 (41,800 - 79,600)	13,000 (9,180 – 17,600)	10,700 (7,550 - 14,500)	11,600 (8,160 - 15,700)	8,310 (5,920 - 11,100)	12,700 (9,120 - 17,500)
Global	2,230,000 (1,040,000 – 3,330,000)	261,000 (136,000 – 364,000)	212,000 (114,000 – 292,000)	290,000 (192,000 – 386,000)	323,000 (230,000 – 430,000)	675,000 (428,000 – 899,000)

Table S11 – Premature PM2.5-related mortality (IHD+Stroke+COPD+LC) in ten world regions, and contributions from different sectors (deaths in 2005), showing the deterministic mean estimates. All numbers are rounded to three significant digits.

	All Anthr.	All Transp.	Land Transp.	Energy	Industry	Resid. & Comm.
NA	76,300	47,300	41,800	27,900	18,500	16,600
SA	24,800	7,980	7,500	1,930	12,500	5,390
Europe	251,000	94,700	66,000	42,800	28,900	68,200
FSU	222,000	32,500	28,400	40,000	22,200	124,000
Africa	29,000	2,130	1,690	3,000	2,180	12,000
India	458,000	24,800	22,600	44,800	41,100	197,000
East Asia	1,110,000	63,200	49,700	123,000	186,000	233,000
SE Asia	124,000	15,300	12,800	21,900	26,000	63,100
Australia	276	195	141	189	216	165
ME	66,200	13,800	11,400	12,100	8,930	14,700
Global	2,360,000	302,000	242,000	317,000	347,000	734,000

Table S12 – Premature $PM_{2.5}$ -related mortality (IHD+Stroke+COPD+LC) per million people in total population in ten world regions, and contributions from different sectors (deaths per million people in 2005), showing the mean from 1000 Monte Carlo simulations. All numbers are rounded to the nearest unit.

	All Anthr.	All Transp.	Land Transp.	Energy	Industry	Resid. & Comm.
NA	169	62	54	38	25	22
SA	77	20	19	5	33	14
Europe	457	149	109	74	50	124
FSU	714	102	90	126	72	384
Africa	32	2	2	3	2	14
India	268	15	14	27	25	118
East Asia	683	39	31	76	115	144
SE Asia	186	23	19	31	38	93
Australia	33	11	7	10	12	8
ME	96	21	17	19	13	20
Global	321	38	31	42	47	97

Table S13 – Premature ozone and $PM_{2.5}$-related mortality in ten world regions for the contribution of all anthropogenic emissions at 2.5°x1.9° horizontal resolution (deaths in 2005), showing the deterministic mean estimates. All numbers are rounded to three significant digits.

	Ozone respiratory mortality	$PM_{2.5}$ mortality (IHD+Stroke+COPD+LC)
NA	36,500	116,000
SA	9,470	21,000
Europe	32,400	337,000
FSU	9,930	308,000
Africa	9,060	33,600
India	163,000	478,000
East Asia	171,000	1,230,000
SE Asia	29,700	134,000
Australia	862	468
ME	18,600	80,600
Global	480,000	2,740,000

Table S14 – Premature ozone and $PM_{2.5}$-related mortality in ten world regions for the contribution of all anthropogenic emissions for the simulations at 0.67°x0.5° regridded to 2.5°x1.9° horizontal resolution (deaths in 2005), showing the deterministic mean estimates. All numbers are rounded to three significant digits.

	Ozone respiratory mortality	$PM_{2.5}$ mortality (IHD+Stroke+COPD+LC)
NA	35,700	57,900
SA	9,700,	10,200
Europe	32,500	241,000
FSU	10,000	197,000
Africa	9,470	25,500
India	159,000	435,700
East Asia	173,000	1,049,000
SE Asia	30,200	98,100
Australia	820	221
ME	18,600	59,300
Global	479,000	2,174,000

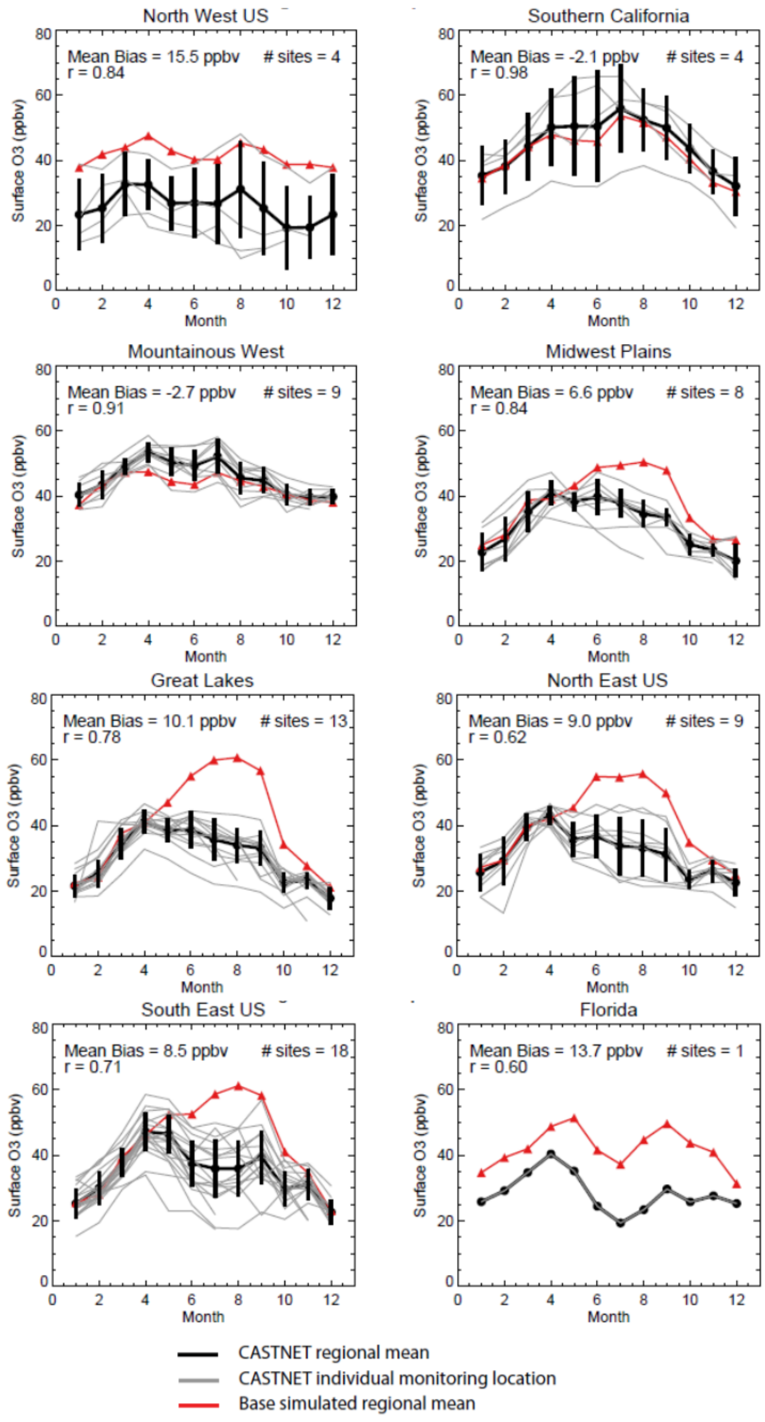

Figure S1 – Comparison of simulated monthly mean surface ozone concentrations (2005) with CASTNet monitored concentrations (2005) for eight US regions, showing modeled regional mean (red), CASTNET regional mean (black) and individual monitoring locations (grey). An overall model bias of 7.2 ppbv is calculated across all stations.

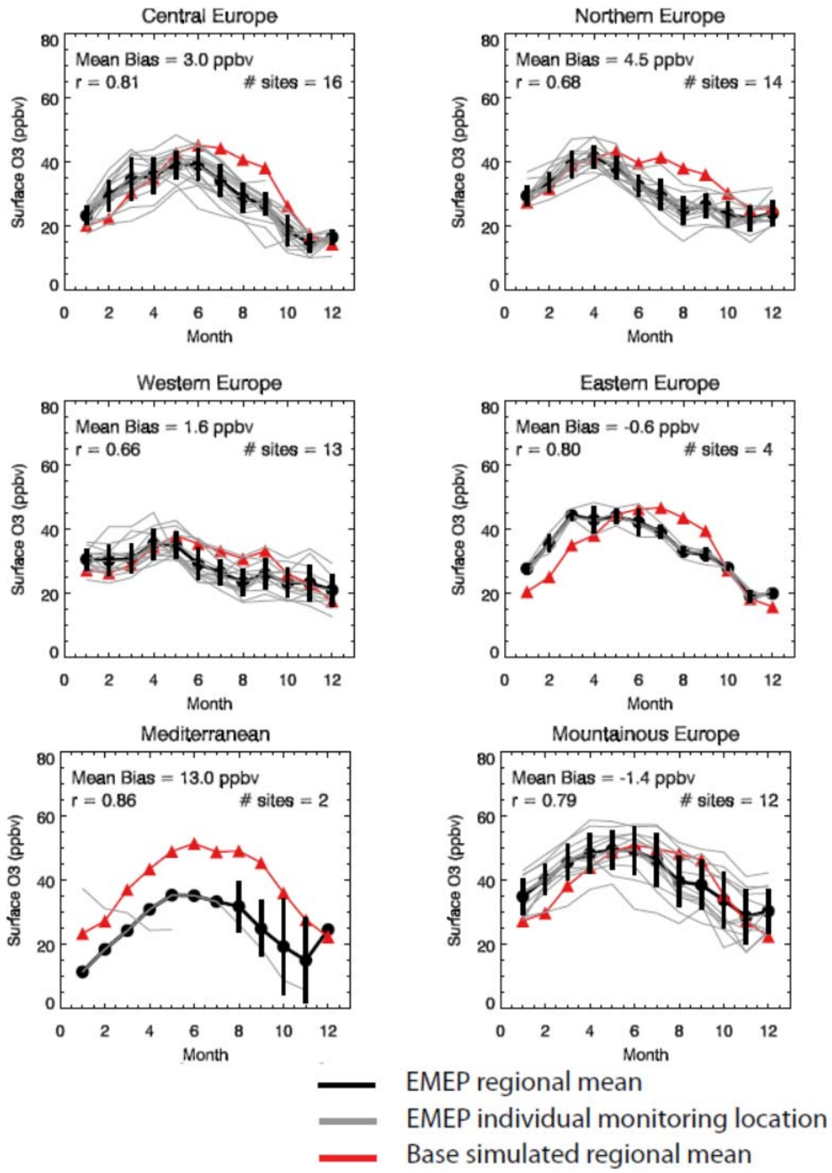

Figure S2 – Comparison of simulated monthly mean surface ozone concentrations (2005) with EMEP monitored concentrations (2005) for six European regions, showing modeled regional mean (red), EMEP regional mean (black) and individual monitoring locations (grey). An overall model bias of 2.3 ppbv is calculated across all stations.

(A) IMPROVE

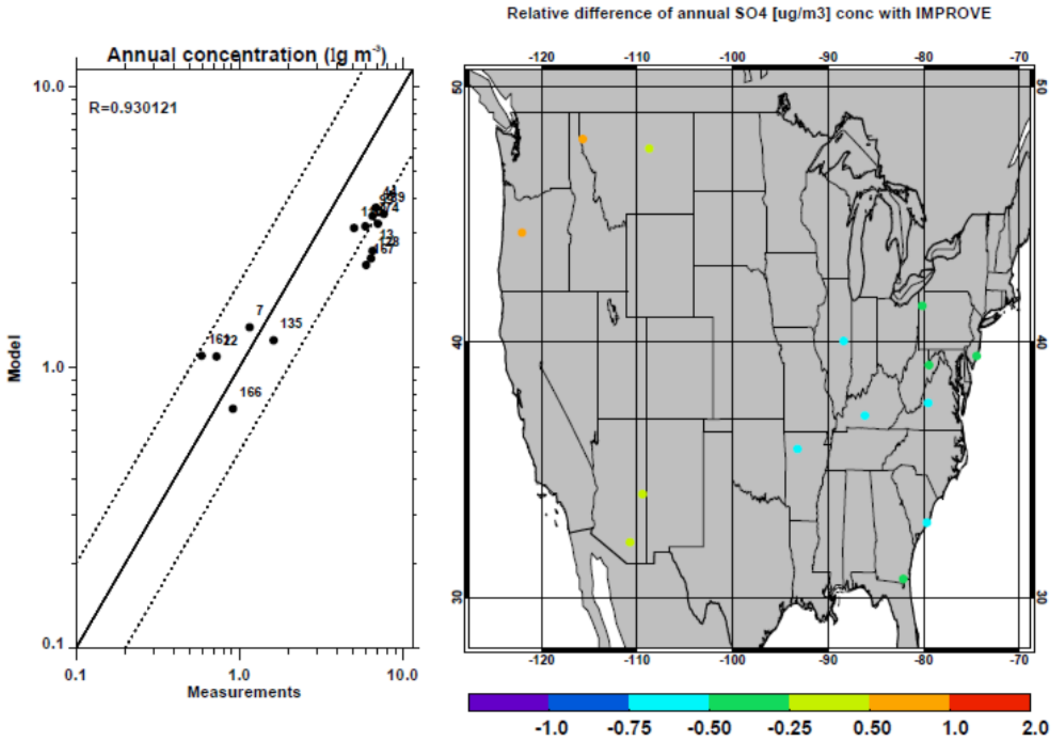

(B) EMEP

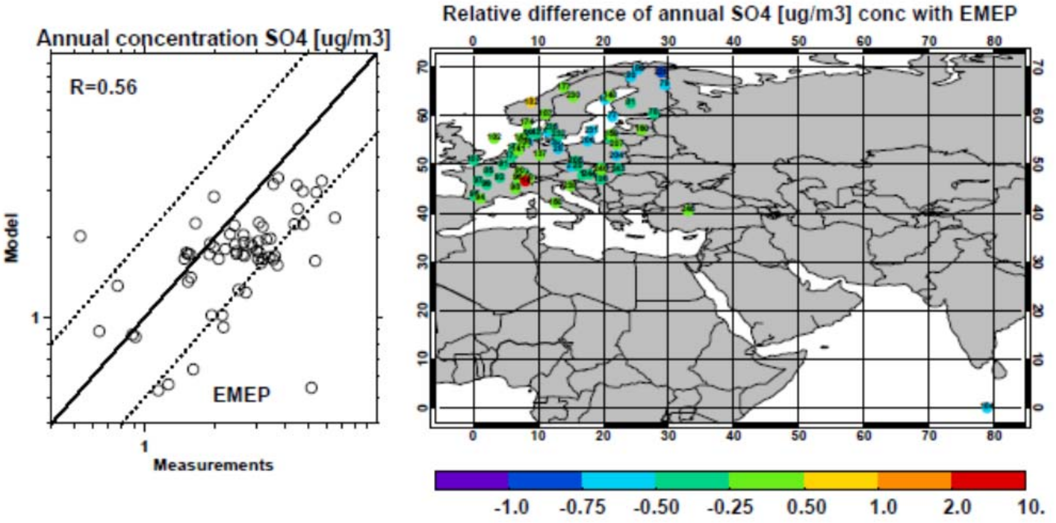

Figure S3 – Comparison of the simulated annual average (2005) surface SO_4 concentrations (μg m^{-3}) with annual average (2005) observations from the IMPROVE(A) and the EMEP (B) surface monitoring networks for the US and Europe, respectively. The left panels show modeled versus observed concentrations (μg m^{-3}) with the dashed 1:2 and 2:1 lines representing

agreement within a factor of 2. The right panels show a map of [(modeled-observed)/observed] values.

(A) IMPROVE

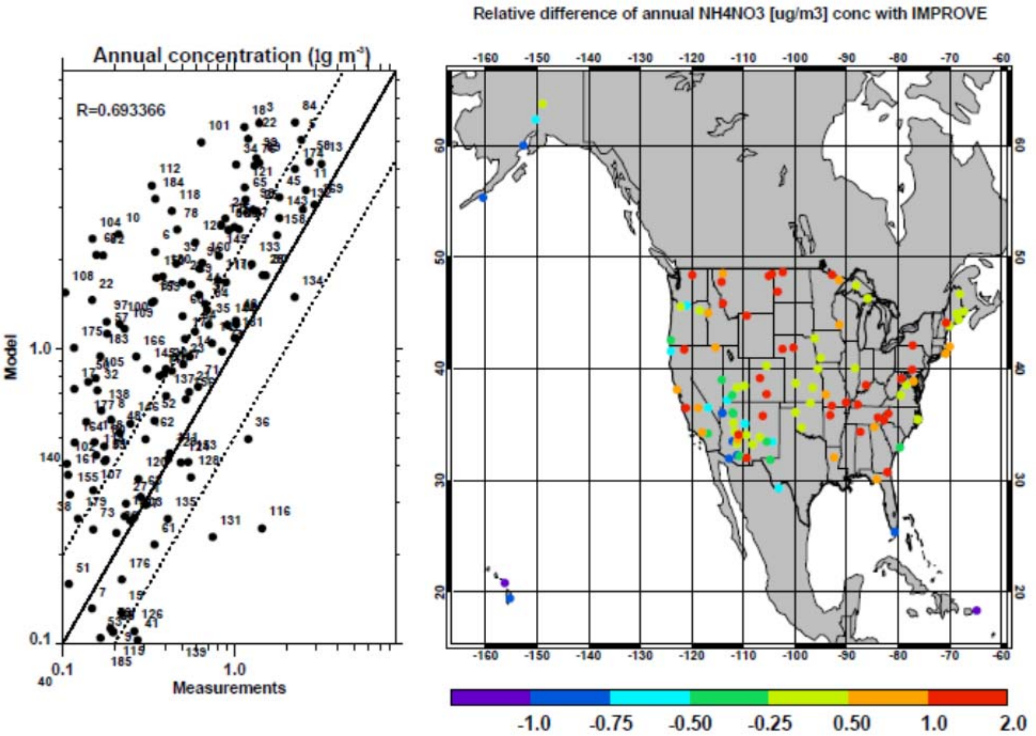

(B) EMEP

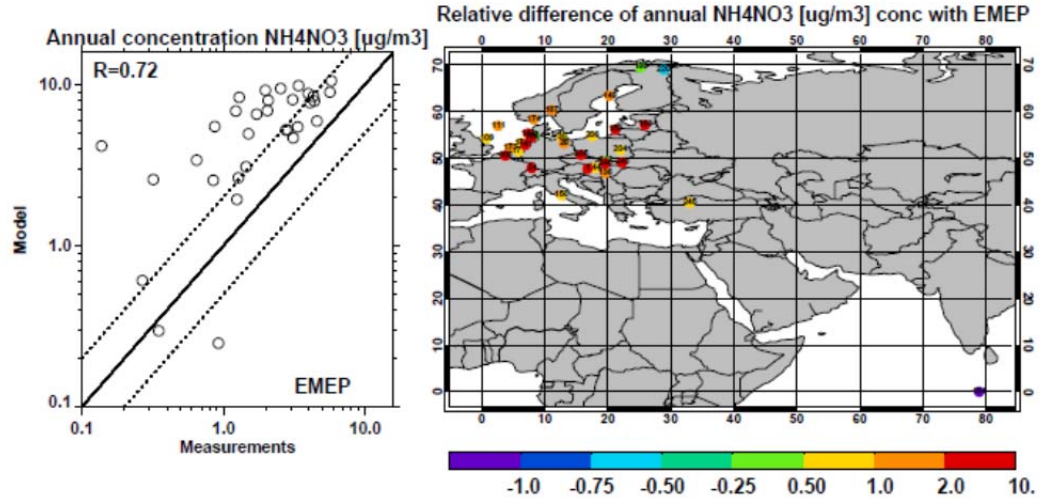

Figure S4 – Comparison of the simulated annual average (2005) surface NH_4NO_3 concentrations ($\mu g\ m^{-3}$) with annual average (2005) observations from the IMPROVE (A) and the EMEP (B)

surface monitoring networks for the US and Europe, respectively. The left panels show modeled versus observed concentrations (µg m^{-3}) with the dashed 1:2 and 2:1 lines representing agreement within a factor of 2. The right panels show a map of [(modeled-observed)/observed] values.

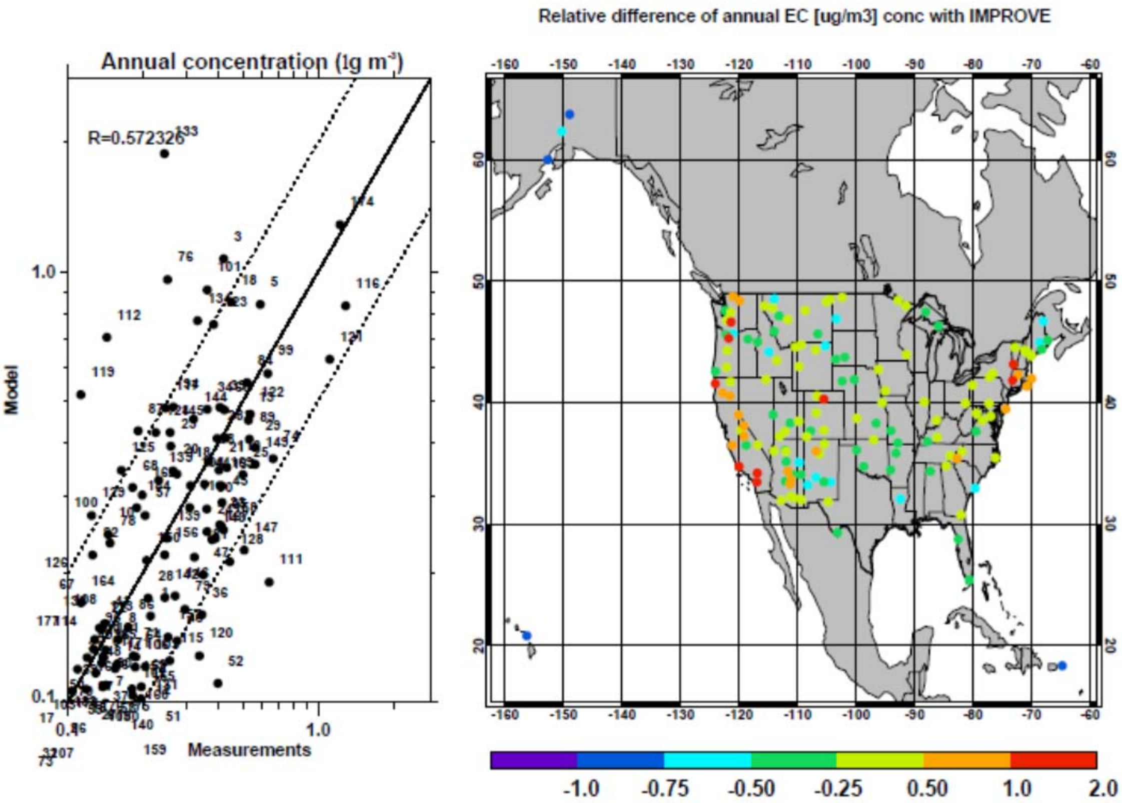

Figure S5 – Comparison of the simulated annual average (2005) surface EC concentrations (µg m^{-3}) with annual average (2005) observations from the IMPROVE surface monitoring networks, US. The left panels show modeled versus observed concentrations (µg m^{-3}) with the dashed 1:2 and 2:1 lines representing agreement within a factor of 2. The right panels show a map of [(modeled-observed)/observed] values.

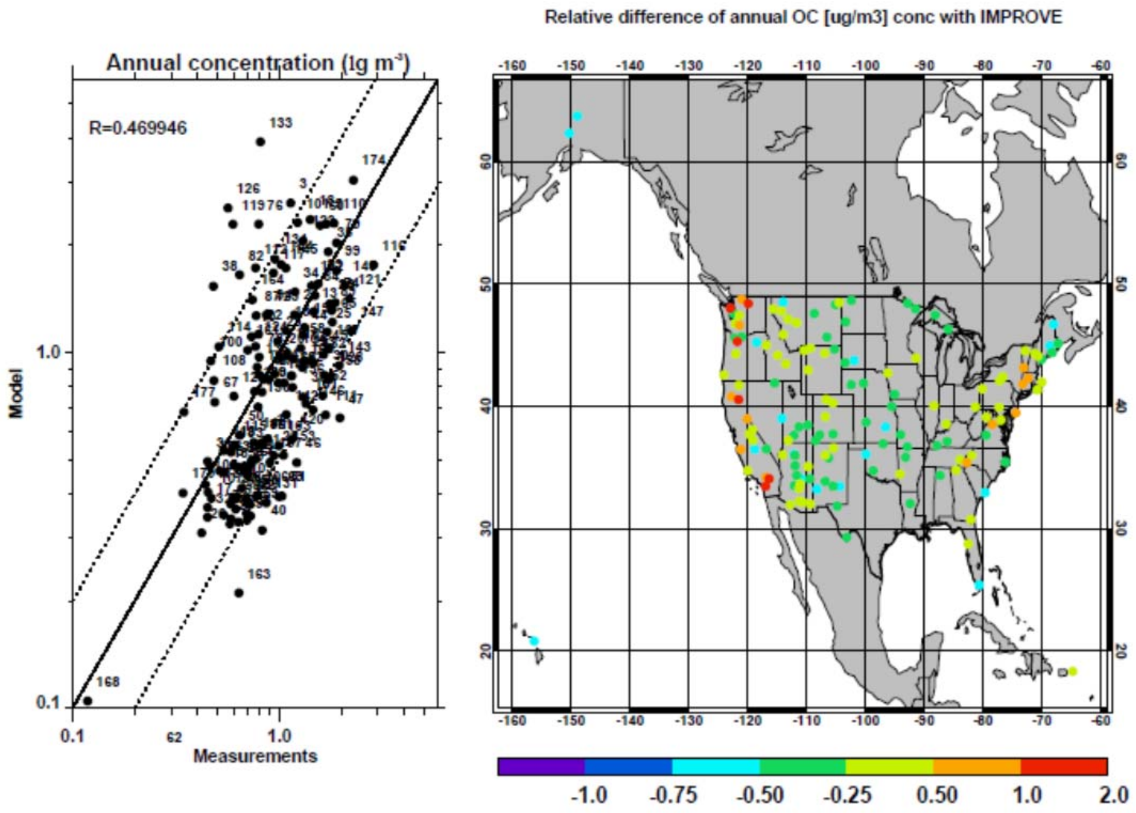

Figure S6 – Comparison of the simulated annual average (2005) surface OC concentrations (µg m^{-3}) with annual average (2005) observations from the IMPROVE surface monitoring networks, US. The left panels show modeled versus observed concentrations (µg m^{-3}) with the dashed 1:2 and 2:1 lines representing agreement within a factor of 2. The right panels show a map of [(modeled-observed)/observed] values.

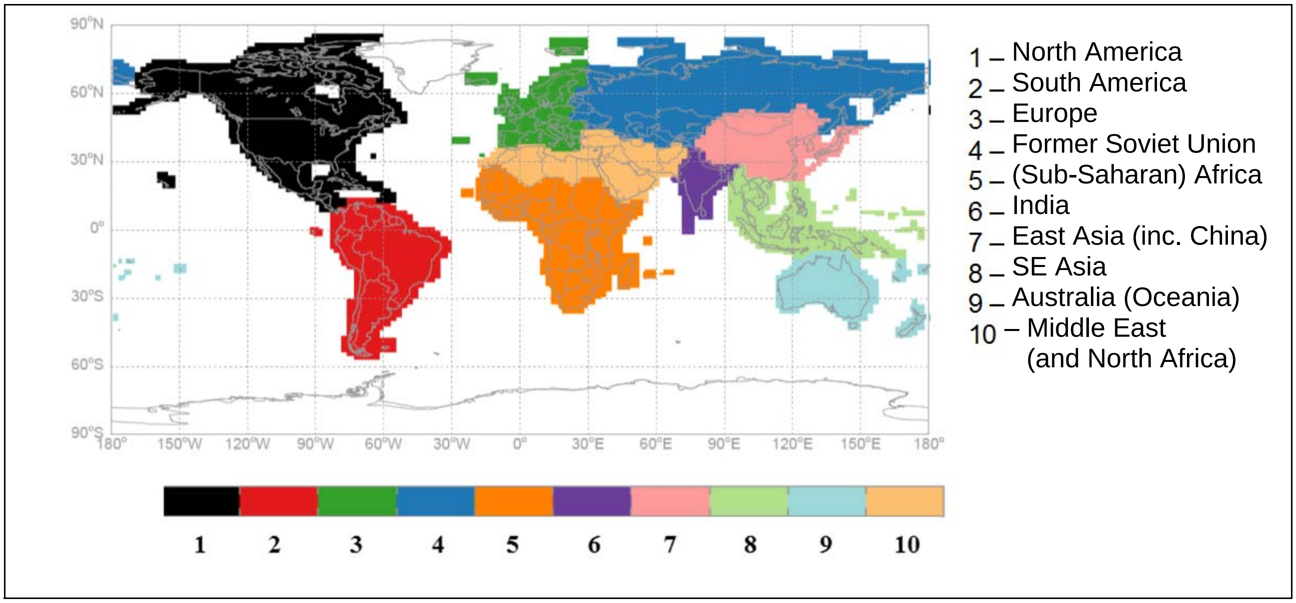

Figure S7 – Ten World Regions.

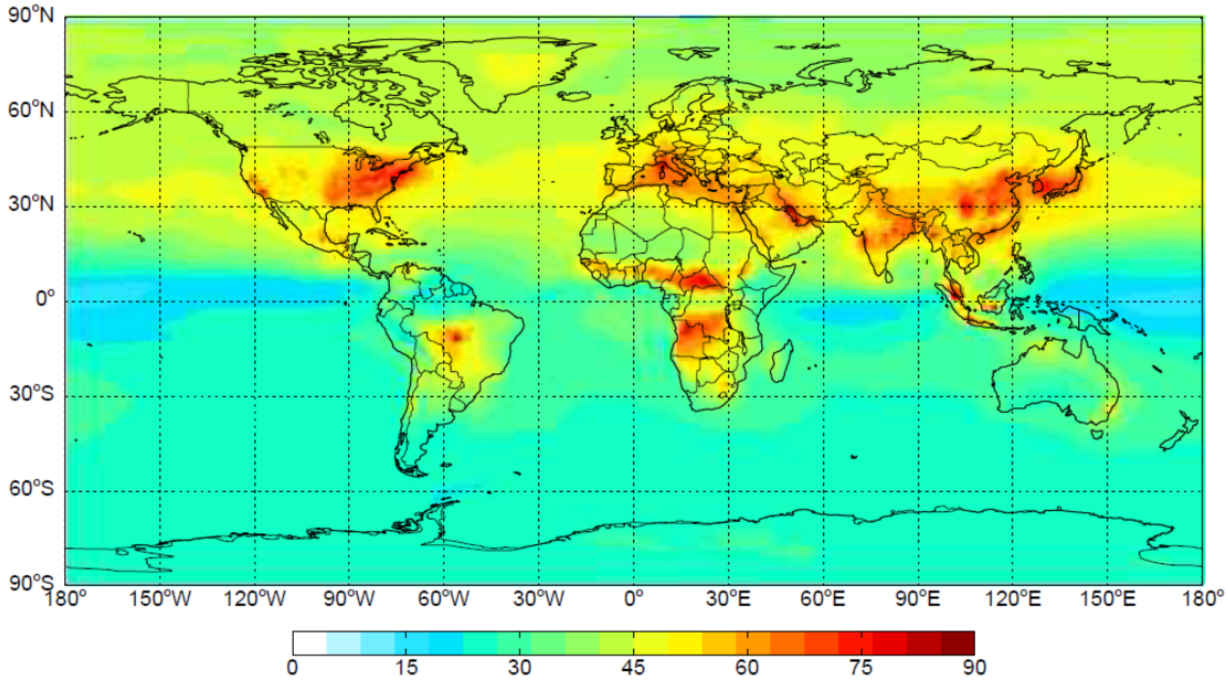

Figure S8 – Ozone concentrations in the baseline simulation (ppb), showing the average 1-hr daily maximum ozone for the consecutive 6-month period with the highest average in each cell.

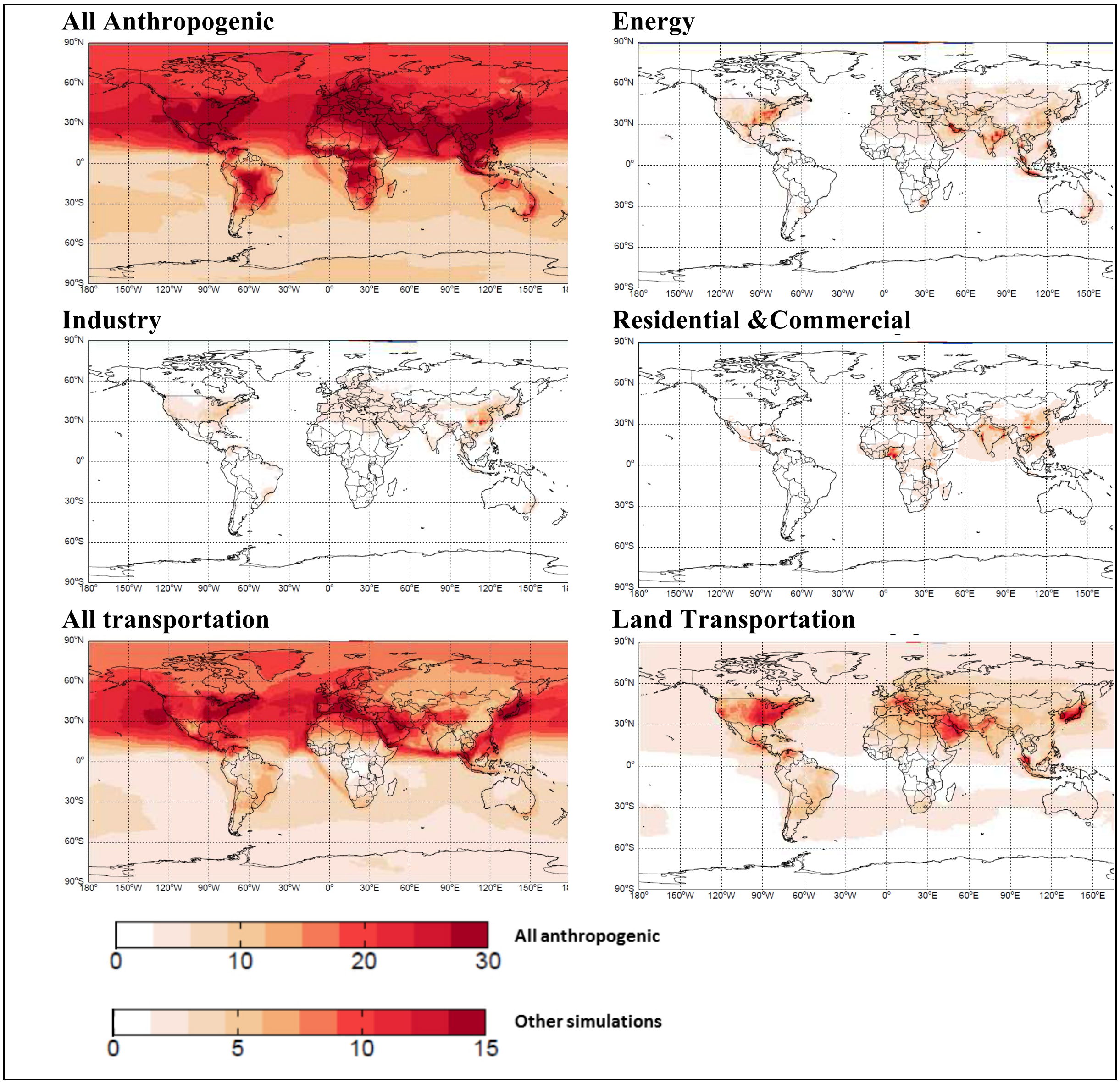

Figure S9 – Difference in ozone concentrations between the baseline and zeroed-out simulations (ppb), using the average 1-hr daily maximum ozone for the consecutive 6-month period with the highest average in each cell.

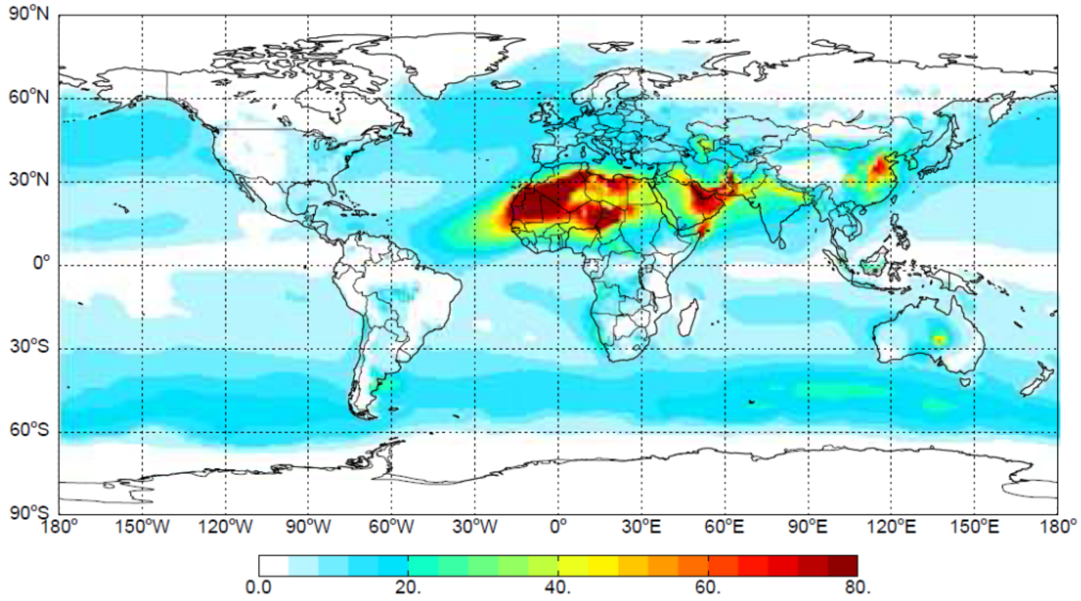

Figure S10 – $PM_{2.5}$ concentrations in the baseline simulation ($\mu g/m^3$), showing the annual average concentrations in each cell.

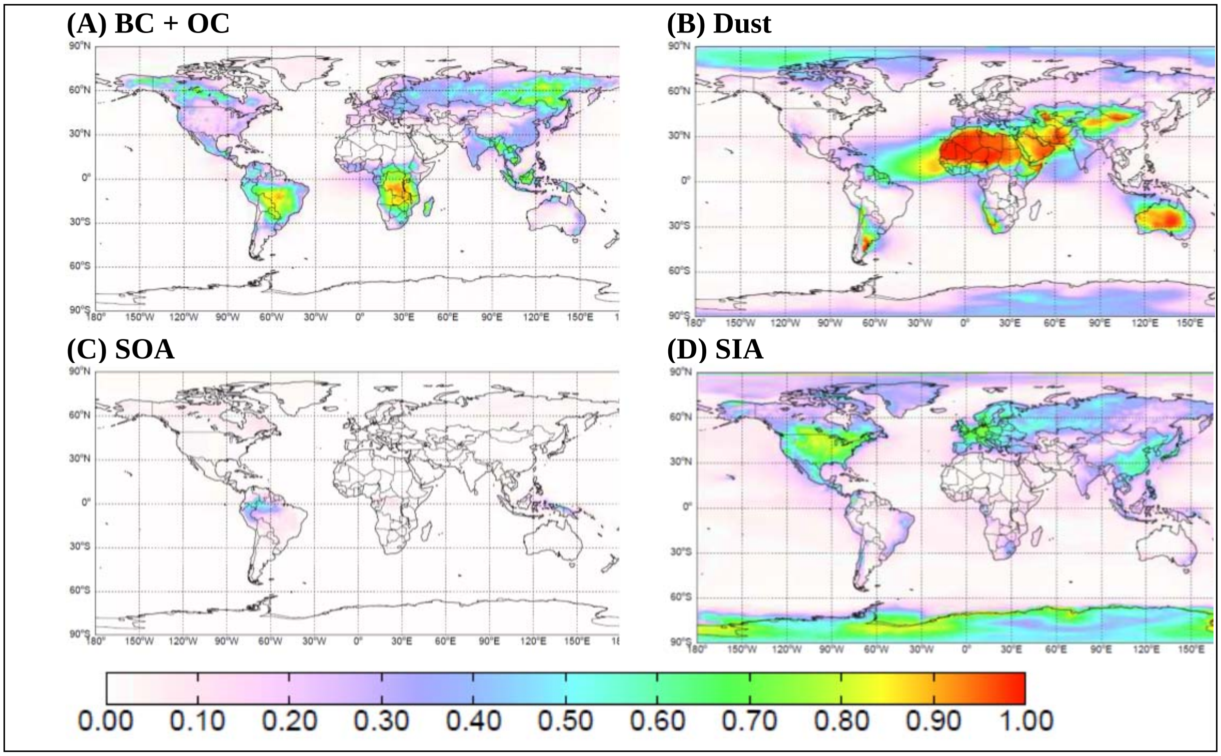

Figure S11 – Fraction of selected $PM_{2.5}$ species (A – D) in total $PM_{2.5}$ concentrations in the baseline simulation.

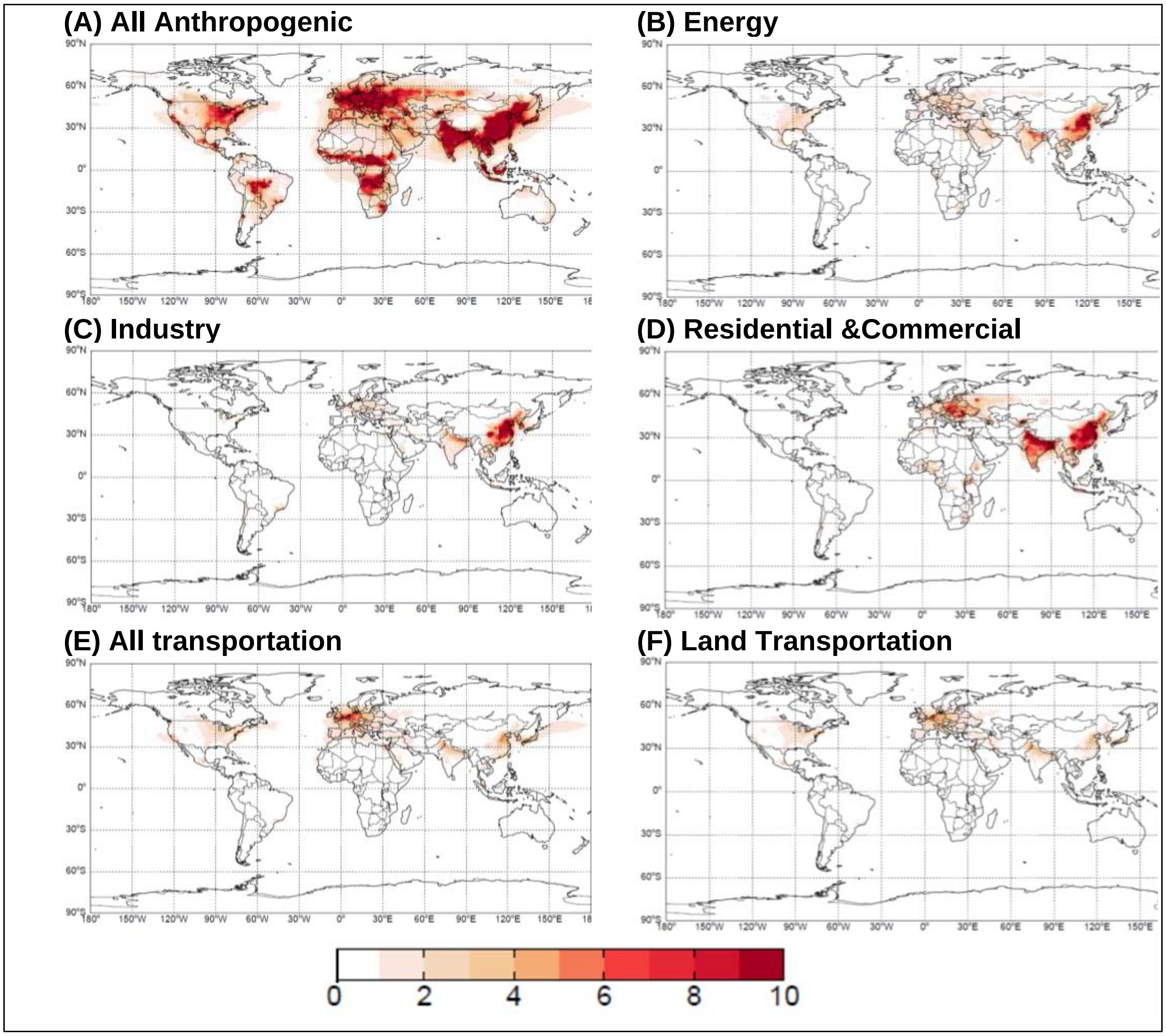

Figure S12 – Difference in $PM_{2.5}$ concentrations between the baseline and zeroed-out simulations (A – F) ($\mu g/m^3$), using the annual average concentrations in each cell.
